# A guide for the deployment, validation, and accreditation of clinical digital pathology tools

**DOI:** 10.1016/j.jpi.2026.100694

**Published:** 2026-07-28

**Authors:** Andrew Janowczyk, Johan Ferrari, Nicolas Brandt, Laura Padayachy, Aurélie Bornand, Suzanne Chartier, Thomas Mallet, Nicolas Roduit, Paul Seed, Medya Tekes Mizrakli, Julien Massonnet, Petros Liakopoulos, Vincent Braunersreuther, Mario Kreutzfeldt, Laura Rubbia-Brandt, Doron Merkler

**Affiliations:** a Department of Biomedical Engineering, Emory University and Georgia Institute of Technology, Atlanta, GA, USA; b Division of Precision Oncology, Geneva University Hospitals, Geneva, Switzerland; c Division of Clinical Pathology, Geneva University Hospitals, Geneva, Switzerland; d Digital Transformation and Augmented Intelligence Department, Geneva University Hospitals, Geneva, Switzerland; e Department of Pathology and Immunology, University of Geneva, Geneva, Switzerland

**Keywords:** Digital pathology, Lab-developed tests, Accreditation, Validation, Clinical deployment, Best-practices

## Abstract

Digital pathology (DP) has become an integral part of modern diagnostic practice, enabling efficient workflows, reproducible assessments, and the integration of computational tools. Whereas many institutions are transitioning toward digital diagnostics, deployment, validation, and routine usage of DP tools in clinical settings remains a major challenge. Existing recommendations often remain high-level, leaving laboratories without detailed, practical guidance on how to structure and document validation processes in real-world settings. To address this gap, we present a framework derived from the experience of Geneva University Hospitals (HUG), which since 2019 has operated a fully digital primary diagnostic workflow processing more than 230,000 slides annually. Drawing on this implementation, we outline best practices and lessons learned for the development, deployment, validation, and accreditation of DP tools. Document templates are also provided, which may serve as adaptable blueprints for other laboratories. This practical guide is intended to support reproducible, transparent, and audit-ready validation processes, helping institutions bridge the gap between general recommendations and operational deployment of DP tools in accredited diagnostic environments.

## 1 Introduction

Digital pathology (DP) represents a transformative shift in the field of pathology, where traditional methods of analyzing tissue samples using glass slides and microscopes are replaced with digital high-resolution images and potentially augmented by computational analysis.^1^ The digitization of histopathological specimens facilitates their retrieval, evaluation, and sharing while enabling advanced computational tools to enhance the efficiency and reproducibility of diagnostic processes.^2^, ^3^ These tools support pathologists by quantitative and objective quality control,^4^, ^5^ automating routine tasks like quantification,^6^, ^7^, ^8^ and prioritizing diagnostically relevant slides for review.^9^, ^10^ Furthermore, DP opens new avenues for personalized medicine by enabling the reproducible extraction of quantitative data for image-based biomarkers, thus improving diagnostic accuracy, guiding treatment decisions, and monitoring of treatment response.^11^, ^12^, ^13^, ^14^, ^15^

However, before using internally developed DP tools in a routine clinical workflow, their validation and accreditation remain paramount in ensuring the reliability, accuracy, and consistency of diagnostic processes. To begin, it is crucial to differentiate between *qualification*, *validation*, and *accreditation* processes.^16^ *Qualification* pertains to verifying that equipment and systems are correctly installed and functioning according to specified requirements, encompassing stages such as design qualification (DQ), installation qualification (IQ), operational qualification (OQ), and performance qualification (PQ).^17^ Once the system has been qualified, *validation* focuses on ensuring that the entire process in question (e.g., creating diagnostic grade whole-slide images (WSIs)) consistently meets its intended purpose and predetermined specifications.^18^ This involves confirming that, e.g., tools produce reliable, accurate, and consistent results in their application. Lastly, *accreditation* broadly implies evaluation and certification of lab practices, personnel competence, equipment performance, and quality management systems against established standards and guidelines, which adhere to stringent quality control measures, ensuring the integrity and traceability of data from specimen collection to reporting.^19^ It is important to note that a lab *cannot accredit itself;* only an external auditor may provide formal approval of the completed validation and confirm it complies with current regulations (e.g., in Switzerland the Swiss Accreditation Service (SAS),^h^ in Germany the Deutsche Akkreditierungsstelle (DAkkS),^i^ and in the US, the internationally recognized College of American Pathologists (CAP) Accreditation program^j^). Thus, in summary, *qualification* is specific to the operational readiness of equipment, *validation* encompasses broader assessment of the process's effectiveness, and an external accreditor certifies the validation process.

Of note, whereas an accredited lab workflow encompasses all validated steps from specimen preparation to diagnostic reporting, individual components such as DP tools may not themselves need to be independently accredited. In practice, accreditation applies to the controlled use of these tools within the lab process rather than to the tool's internal logic or algorithmic design. For instance, a laboratory may develop and validate a DP tool as a Laboratory Developed Test (LDT),^20^, ^21^, ^22^ demonstrating its reliability, accuracy, and reproducibility within the intended accredited workflow. Nevertheless, the tool in isolation is itself not formally accredited, as accrediting bodies typically do not certify proprietary software; they instead evaluate the laboratory's implementation, documentation, quality control measures, and adherence to regulatory standards. Consequently, a tool can be safely employed in clinical practice under an accredited workflow provided that its validation is rigorously documented, but its standalone accreditation would require separate regulatory approval, such as through medical device clearance or compliance with software-specific standards.^23^ In this manuscript, care has been taken to precisely use these terms, with the phrase “validation/accreditation” used when the choice of terminal state is left to the discretion of the institution.

That said, tools often begin as LDTs with the intent of being developed and validated for use *within a specific lab* adhering to local or national standards (e.g., ISO 15189:2022^24^) where they undergo regular audits and evaluations to ensure accuracy and reliability. Once mature, institutions and companies often want to commercialize them for *broader multi-site use*, and thus must comply with the European Directive 98/79/EC^k^ or Regulation (EU) 2017/746^25^^,^^l^ or FDA regulations in the United States.^26^, ^27^ As such, the main difference between an LDT and a CE + IVDR (Conformité Européenne - In Vitro Diagnostic) or FDA-approved technique lies primarily in their regulatory pathways and oversight requirements. Beyond LDT requirements, CE-marked IVDs undergo conformity assessment procedures under the IVDR, which typically involve a notified body for higher-risk devices (Classes B, C, and D) that conducts quality management system audits and technical documentation reviews. FDA-cleared IVDs follow either the 510(k) pathway^28^, ^29^ requiring demonstration of substantial equivalence to predicate devices through analytical performance studies, or the premarket approval pathway^30^ for high-risk devices requiring clinical trial data. Importantly, regardless of regulatory approval status, all diagnostic tests—including CE-marked IVDs and FDA-cleared devices—must undergo local verification or validation before clinical implementation to confirm they perform reliably within the specific context of local laboratory conditions, sample types, and clinical workflows.^31^

Despite growing adoption of DP,^32^ standardized implementation of validation protocols varies markedly across institutions, an issue that may in part stem from the fact that most available guidance remains at a high-level rather than providing detailed, prescriptive procedures.^33^ This guide aims to address that gap by presenting practical real-world guide based on insights from Geneva University Hospitals (HUG), which completed its transition to a fully digital primary diagnostic workflow begun in 2019, and now develops and deploys in-house tools for routine use. Drawing on HUG's experience processing over 1500 slides daily (230,000+ annually), this article outlines key considerations, challenges, and best practices for tool validation and accreditation. Document templates are provided in the appendix that can serve as blueprints to further facilitate implementation of the processes outlined.

### 1.1 Roles and responsibilities

Personnel engaged in the tool creation, deployment, validation, and accreditation process can be defined by their roles. Depending on the size of the institution, these roles may be assigned several people, or conversely, one person may be assigned to several roles. At HUG, the following roles were defined:

#### Technical roles

Algorithm researcher (*AR*): The *AR* is responsible for developing specific technologies, models, or algorithms. This role may be undertaken, for example, by: (a) a student working on a terminal project (e.g., a Master's or PhD thesis), (b) an in-house/consulting professional software developer, or (c) an external third-party institution. Depending on the context, *AR* may thus be temporary, permanent, or externally contracted.

Deployment engineer (*DE*): The *DE*'s role involves transforming an *algorithm* developed by e.g., *AR*, into a robust, production-ready *tool* suitable for real-world clinical use—a process known as *hardening*. In practice, hardening involves optimizing the algorithm for performance and reliability, implementing testing and error-handling mechanisms, ensuring compliance with security and privacy standards, and packaging it for integration within hospital systems. The *DE*'s responsibilities include performing the hardening, as well as, developing necessary connectors (e.g., standardized software development kits, SDKs) to interface with existing infrastructure and deploying the tool within the IT environment. Typically, the *DE* is a hospital employee and, unlike *AR*, assumes long-term responsibility for the tool's maintenance and sustainability.

IT staff (*IT*): *IT* staff concentrate on the broader technical environment, ensuring necessary hardware and software systems (e.g., high-performance computing solutions) support the tool's deployment and ongoing operation. They need to ensure that underlying technical infrastructure is robust, secure, and compliant with regulatory standards. This includes maintaining network stability, managing storage solutions, and ensuring cybersecurity measures protecting patient data.

#### Medical roles

Experimental pathologist (*EP*): *EP*s collaborate closely with *AR*s and *DE*s to define scope and requirements of the tool, ensuring it addresses relevant clinical challenges. As domain experts, they help identify relevant cases/slides, and provide feedback/annotations as needed. Acting as “beta testers,” they rigorously evaluate the algorithm during its development phase, providing essential feedback to refine and enhance its performance. Their clinical expertise and hands-on involvement help bridge the gap between theoretical development and practical application, ensuring the final tool is clinically viable, user-friendly (ergonomic), and relevant.

Routine pathologist (*RP*): *RP*s are the intended end-users of the tool who will use it in daily practice. Unlike *EP*s, they were not actively involved in the tool's development. Instead, they are exposed to it only after it: (a) has been fully integrated in their routine clinical workflows and validated and (b) they have received training on how to use it. As such, effectiveness and user-friendliness of the tool in real-world scenarios are paramount to them.

#### Laboratory roles

Laboratory technician (*LT*): *LT*s assure the day-to-day operations of the lab, executing tests, maintaining equipment, troubleshooting issues, and ensuring accurate results. When new tools are introduced, e.g., via hand-off from *DE*, they are responsible for supporting (e.g., diagnostic tools) and/or applying (e.g., quality control tools) them in daily workflow, following protocols and guidelines established by *DE* and Lab managers. By adhering to quality control standards and providing feedback on performance and usability, they ensure that integration of new tools is in line with the lab's needed efficiency and reliability.

Laboratory manager (*LM*): *LM*s oversee overall operations of the lab, ensuring that all processes run smoothly and efficiently. In the context of implementing new tools, they are responsible for coordinating between all the various stakeholders listed here. They ensure that the lab is equipped with the necessary resources and infrastructure to support the new technology. Additionally, they oversee compliance with regulatory standards, manage quality control, and ensure that staff are adequately trained to use new tools effectively.

Departmental leadership (*DL*): They provide strategic direction and oversight for the lab's operations. In the context of implementing new tools, they play a pivotal role in road-mapping, decision-making, resource allocation, and driving organizational change. *DL* ensures that the development and adoption of new technologies aligns with institutional goals, guidelines, and objectives. They facilitate communication and coordination among various departments, including research, clinical, and administrative teams, to ensure a smooth integration process. Additionally, they champion innovation and quality improvement initiatives, encouraging a culture of continuous learning and adaptation.

#### Regulatory roles

Quality officer (*QO*): A hospital employee ensuring compliance with internal standards and external accreditation requirements. They serve as liaison between the hospital and accreditation bodies, maintaining transparency and upholding the integrity of the hospital's quality management system.

Accreditors (*AC*): Representatives of the external body responsible (e.g., SAS, CAP) for evaluating and certifying that the lab, including its processes and tools meet industry standards and regulatory requirements. Their role involves conducting thorough audits of the lab's processes, equipment, personnel, and documentation.

### 1.2 High-level overview

The high-level overview of the validation/accreditation process (see Fig. 1) provides a structured framework for understanding the key stages involved. Each process box corresponds to a numbered section below for easy reference and comprehension. The workflow is delineated into four distinct phases, described below, represented by different colors.

**Fig. 1:**
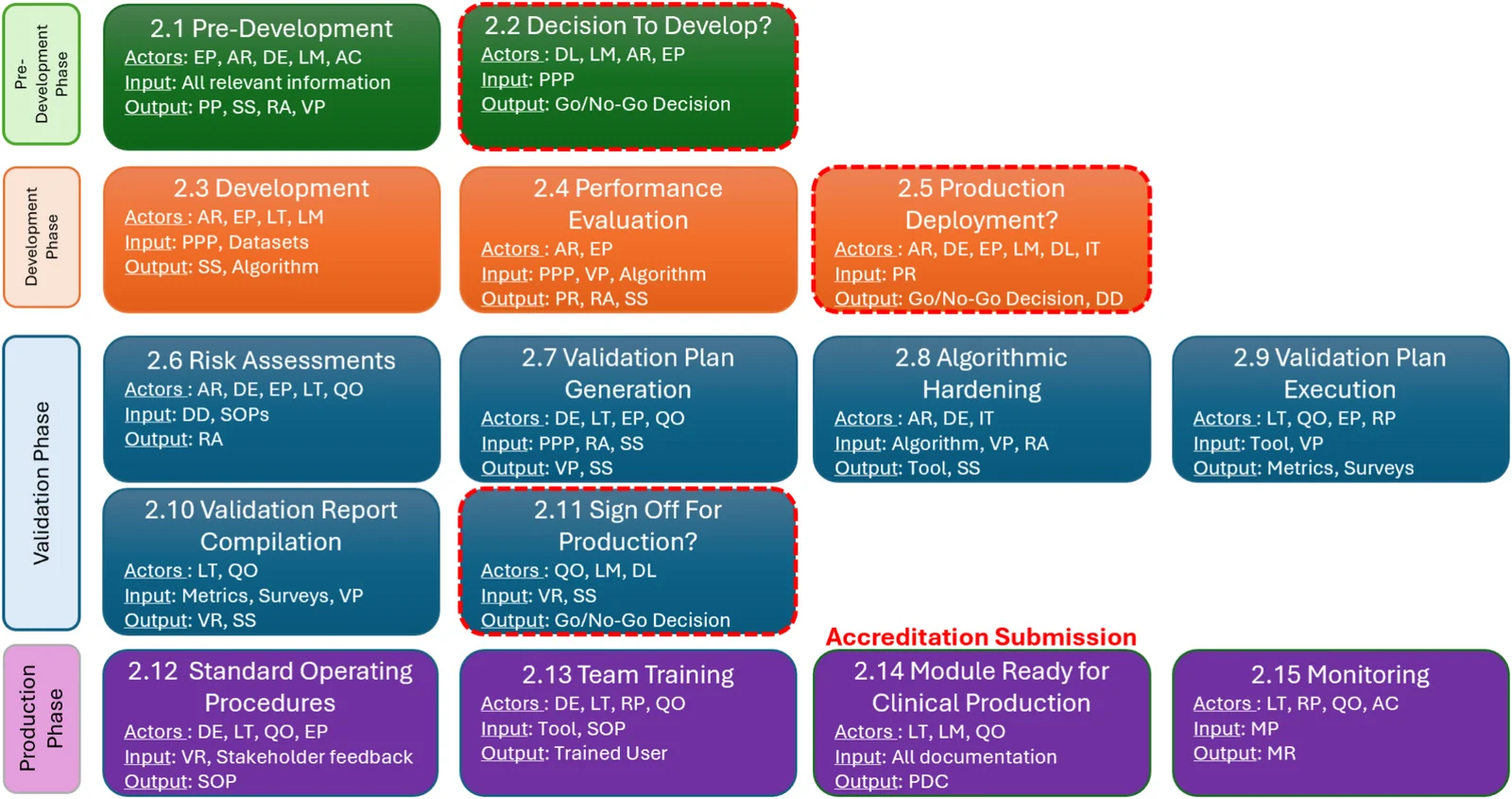
High-level overview with each stage coinciding with a numbered sub-section in the main text below for easy reference. The workflow is broken into four phases, as indicated by the different colorings. Pre-development Phase and Development Phase, occur pre-validation/accreditation, wherein the algorithms are designed and developed. The Validation Phase sees the now hardened tool validated and deployed for routine clinical usage. Lastly, the Production Phase is a process of continual assessment and monitoring, as defined by the validation/accreditation needs, to ensure long-term stability and effectiveness of the deployed tool. Stages surrounded by a red dashed border indicate decision points. Additional acronyms: Decision Document (DD), Monitoring Plan (MP), Monitoring Report (MR), Production Deployment Checklist (PDC), Project Plan (PP), Project Plan Package (PPP), Performance Report (PR), Risk Assessment (RA), Standard Operating Procedures (SOPs), Software Specification (SS), Validation Plan (VP), Validation Report (VR). (For interpretation of the references to color in this figure legend, the reader is referred to the web version of this article.)

The Pre-Developmental Phase determines if criteria such as feasibility and expected added value are sufficient to justify moving to the developmental phase, and yields a precise *Project Plan Package*. It can help to think of this package as a folder containing individual named documents/deliverables, as well as supporting literature, diagrams, schedules, or planning documents (e.g., Gantt charts). As such, in the Developmental Phase, the algorithm is designed, developed, and evaluated according to that *Project Plan Package*. Establishing a realistic timeline—with clear milestones, deadlines, and checkpoints—is essential for an effective developmental phase, and can be guided by best practices from software and research projects, such as Agile and Scrum methodologies^34^, ^35^ that emphasize phased progress, collaboration, and continuous feedback and improvement. Next, the Validation Stage ensues, wherein algorithms are forged into hardened tools, which undergo rigorous validation and deployment for routine clinical usage. The Production Phase signifies an ongoing process of assessment and monitoring post-deployment. This phase is essential for maintaining long-term stability and effectiveness of deployed algorithms, incorporating quality control measures, regular maintenance and audits, along with feedback mechanisms to address evolving validation/accreditation needs.

### 1.3 Introduction to worked HPyloriDet example

To provide real-world context for the various stages, our *Helicobacter pylori* (HP) detection tool (HPyloriDet) is used as an example. Briefly, HPyloriDet is a deep learning-based tool, which analyses HP-stained slides (IHC, DAB), and integrates outputs into Sectra for pathologist review (see Table 1 and Fig. 2). This tool was deployed as a beta version in April 2024, and then fully clinically validated since March 2025. During that time, it has processed approximately 6000 and 1700 WSIs, respectively (*manuscript under preparation*).

**Table 1:** Overview of the HPyloriDet Project.

Aspect	Summary
Purpose	Development and clinical deployment of a deep learning-based screening tool for detecting *Helicobacter pylori* on immunohistochemically stained whole-slide images (WSIs), integrated directly into the routine digital pathology workflow.
Clinical setting	Geneva University Hospitals (HUG), fully integrated diagnostic workflow with the Sectra digital pathology platform.
Study datasets	Approximately, 1129 IHC WSIs broken down into smaller, potentially overlapping, subsets: -Initial training with manual annotations: 20 WSIs -Patch enrichment during retraining: 630 patches from 427 WSIs -Slide-level training / control handling training: 132 WSIs -Slide-level validation/control handling validation: 103 WSIs-time gain evaluation: 50 WSIs -First questionnaire: 123 WSIs -Second questionnaire: 129 WSIs -Final questionnaire: 172 WSIs -Run time analysis: 198 WSIs -Integration tests/repeatability/reproducibility/resource usage evaluation: 25 WSIs
Annotation effort	23,000+ image patches generated from expert pathologist annotations, including explicit handling of ambiguous-positive regions to minimize false negatives.
Model architecture	DenseNet-based patch classifier combined with a slide-level naïve Bayes aggregation model.
Clinical deployment	Automatically analyzes approximately 100 slides per week within routine practice.
Patch-level performance	Accuracy 94%, sensitivity 93%, specificity 94%, NPV 99% on the internal validation dataset.
Slide-level performance	Internal validation accuracy 98%, sensitivity 100%, specificity 98%.
Clinical impact	Mean diagnostic review time reduced by 26% (36.2–26.7 s per case), reported improved ergonomics and higher confidence when using decision support.
Continuous learning	Supports pathologist feedback collection, routine performance monitoring, retraining using real-world data, and adaptation to external institutions.
Project team	Multidisciplinary collaboration involving pathologists, computational scientists, software engineers, digital pathology specialists, and quality officers, from Geneva University Hospitals (see Section 1.1).

**Fig. 2:**
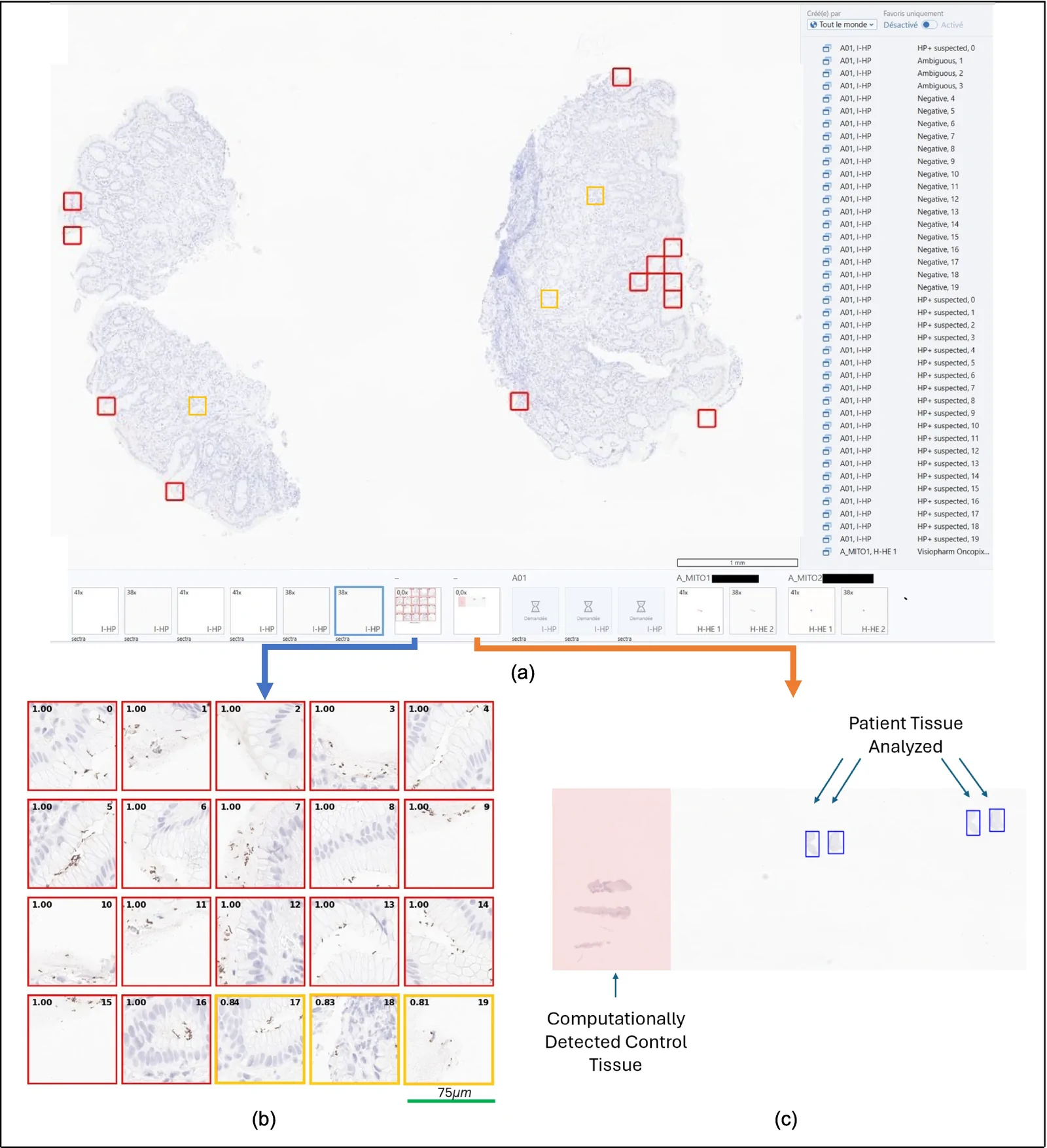
Example of integrated HPyloriDet output into Sectra. (a) Sectra image viewer showing computationally added annotations, colored by confidence (red = high, orange = medium, blue = low), for potential HP-positive regions. They are clickable on the right-hand side, enabling immediate navigation to those regions. (b) Zoomed view of the contact sheet corresponding to the blue arrow in (a), displaying the top 20 highest-probability HP-positive patches. (c) Zoomed view of the item indicated by the orange arrow in (a), the slide overview image, with blue bounding boxes showing where the analysis was performed. The red overlay marks computationally detected control tissue, which was subsequently excluded from analysis. (For interpretation of the references to color in this figure legend, the reader is referred to the web version of this article.)

## 2 The workflow

### 2.1 Pre-development stage

**Scope:** The scope of the pre-development stage encompasses defining objectives, constraints, and requirements of the project. This involves clearly outlining clinical needs, desired outcomes, and target users for the algorithm, but *excludes* any actual tool development until after 2.2 Decision to Develop Stage.

**Participants:** **EP**, **AR**, **DE**, **LM**, **AC.** AC is optional but suggested on first LDT accreditation.

**Input:** These include gathering relevant data, literature reviews, stakeholder input, and existing algorithms or methodologies that may inform the development process, helping to serve as a foundation for defining scope and project requirements.

**Output:** This *Project Plan* includes an overarching summary document containing sections: Background, Motivation, Aims, Timeline, Expected Deliverables (both quantitively and qualitatively in retrospective and prospective validations), and Initial Feasibility Assessments.

Preliminary versions of (a) *Appendix 1 – Software Specification, Responsibility, and Confirmation Plan* (referred to as **“***Software Specification***”** going forward) should be completed, documenting in more detail the expectations of such a tool, its technical requirements, software stack, and assign personnel ownership/responsibility of the various components of its life-cycle to competent personnel, (b) *Appendix 2 - Analysis Of Risks Impacting Work Processes During Major Modifications* (referred to as “*Risk Assessment*”), and (c) *Appendix 3 - Validation Plan and Report* (referred to going forward as “*Validation Plan*” or “*Validation Report*”, as appropriate) with its Section 1 completed. These documents should be updated throughout the validation process until 2.11 Sign Off For Production Stage is completed.

Importantly—these documents are often similar across tools of the same class (e.g., all detection tools, all segmentation tools), and thus benefit from standardization and templating.

Together, the *Project Plan, Software Specification, Risk Assessment*, and *Validation Report* make up what is referred to as the *Project Plan Package.*

**Timeline:** Algorithms with easily interpretable output and clear performance requirements that can be met with current technology, like HPyloriDet, typically require 15–20 h for the pre-development phase (note: unless otherwise stated, all time estimates represent combined hands-on effort from all parties). More sophisticated algorithms, where existing technology may not be readily applicable, or the output less obvious how to interpret (e.g., an image-based biomarker risk score), will likely require substantially more of a time investment.

**Context and considerations:** This stage is potentially the most critical, as it has an extensive impact on the success and completion efficiency of all subsequent stages, and therefore warrants appropriate investment. For example, when the acceptance criteria outlined in the *Validation Plan* are known in advance, relevant elements can be incorporated during the algorithm design and data collection phases, thereby greatly facilitating these processes and avoiding rework.

Appreciating that many tools will be aimed at efficiency and time-gain, it is advisable to record a set of unbiased time measurements—both unaided and aided (e.g., task completion time per minute). Two complementary types of experiments can be considered: (i) a baseline measurement of unaided performance within the image management system (IMS), ideally performed on a larger number of slides, to establish how much room for improvement exists in practice and to provide a benchmark for later aided comparisons; and (ii) a preliminary time-gain experiment, which may be estimated outside the IMS (e.g., using tools such as QuPath^8^), to provide early evidence that tool usage could yield efficiency benefits. It may also be useful to simulate the expected outputs of the tool—for example, by having a second pathologist manually generate a minimal set of results representative of those the tool would ideally produce for the first pathologist to complete their evaluation with. This allows for a controlled estimation of the time or effort differential, even before an algorithm is created. A point of anecdotal evidence suggests that algorithm development may bias the pathologists' interpretative processes; therefore, these measurements should ideally be obtained independently or before the development phase.

Additionally, estimating the annual volume of cases to which the tool will be applicable is crucial for balancing development effort against anticipated benefits (see *Software Specification 4*). If, in practice, the task is completed efficiently without assistance—leaving little room for meaningful improvements at scale—it may be worth reconsidering the development of the tool unless other advantages, such as increased precision/sensitivity or financial benefits, justify its implementation.

To enhance the efficiency of the potential validation process, all collected WSIs—particularly those used during the time measurements—should be retained. Since tool development and validation typically span several months, this period aligns well with the ideal pathology reading washout interval.^36^, ^37^ By preserving WSIs for later use, the same slides can be reused for validation, enabling direct comparisons of evaluation speed and accuracy, thereby increasing statistical power and reliability while minimizing human effort.

Lastly, the scope of the 2.1 Pre-Development Stage should likely be reviewed by the Institutional Review Board (IRB) to ensure ethical compliance, assess potential risks to patients, and oversee proper use of patient data, as responsibility for these aspects ultimately resides with them. It also remains important to emphasize that when the pre-development phase is performed wholly or partially by individuals external to the institution—such as students, contractors, or commercial partners—all data released to them remains subject to institutional regulations (e.g., established data usage agreements), and should be thoroughly anonymized to prevent any linkage between patients, pathology samples, and these pre-development activities.

**Worked HPyloriDet example:** The team was as follows: (i) *EP*: the senior pathologist who proposed the project, (ii) *AR* and *DE* roles were assigned to the same hospital employee, as this was an internal project, (iii) a senior *AR* to supervise the project, with oversight from the (iv) *LM* and *DL*.

The *Project Plan* included a concise background section and literature review on automatic HP detection algorithms, revealing most previous studies focused on non-IHC staining and were not clinically deployed. After a cost–benefit analysis, the primary goal was identified as a clinically deployable screening tool for detecting HP on IHC-stained slides with nearly 100% sensitivity. The key metric in demonstrating value-add was determined to be time-to-diagnosis gain. The user-facing usability/ergonomic requirement (see *Software Specification 7e*) stated that results were to be integrated within the same IMS used during routine diagnostic sign-out for optimal accessibility.

The cohort size needed for developing the tool was estimated to be approximately 100 HP-positive IHC slides scanned at 40× magnification, with 20–30 of them to be annotated by *EP*. The planned baseline approach involved the following steps: (1) tissue detection, (2) identification of regions of interest (ROIs), i.e., those containing putative HP-positive objects, using stain deconvolution, (3) their associated patch extraction, and (4) their classification as positive or negative via deep learning.

The project was divided into three components with the following three deliverables: (1) tissue detection, ROI identification, and patch generation, (2) training the deep learning model focusing on high sensitivity, and (3) hardening and deploying the tool within the existing workflow, ensuring DICOM compatibility and IMS integration, with clear, interpretable outputs presented as either heatmaps highlighting HP-rich areas or direct slide annotations. The latter decision was left open for iterative refinement and consensus-building during development.

The expected duration for these 3 deliverables was approximately 3 weeks, 2 months, and 5 weeks, respectively. The overall 2.1 Pre-Development Stage was completed over the course of 2 weeks.

### 2.2 Decision to develop stage

**Scope:** Review material collected in the 2.1 Pre-Development Stage and decide if development is warranted.

**Participants:** **DL**, **LM**, **AR**, **EP.**

**Input:** The *Project Plan Package* created in the previous stage.

**Output:** A go/no-go decision to advance to the 2.3 Development Stage.

**Timeline**: The decision-making process can likely happen during a single discussion when all necessary documentation is available and reviewed beforehand.

**Context and considerations**: Upon reviewing the *Project Plan Package*, it may become clear that proceeding with tool development is no longer desirable or feasible. Several factors could contribute to this assessment, including: (a) the anticipated improvement over existing clinical methods may be less significant than initially expected; (b) the development plan may prove overly complex, due to unforeseen technical challenges, a broader-than-expected scope, or need for specialized expertise not readily available; (c) the overall available data may be insufficient to support robust training and validation due to a low projected caseload^38^; (d) even if the dataset is large enough for training, rare subtypes (i.e., those part of the long tail^39^) intended for inclusion may be inadequately represented, limiting validation potential; (e) inadequate compute or IT infrastructure to support needed computational throughput or turnaround time; or (f) lack of functionality needed to ergonomically integrate results within the IMS.

Relatedly, once a decision is made to proceed, revisiting the data collection strategy is beneficial. Since the validation set will likely need to include challenging or rare cases—often identifiable only through retrospective and labor-intensive review—prospective/concurrent identification and retention can be facilitated by enabling *EP* and *RP* proactive flagging within the IMS during their routine clinical workflows.

**Worked HPyloriDet example:** *DL* approved the decision to develop upon reviewing the *Project Plan Package*.

### 2.3 Development stage

**Scope:** Focuses on the collaborative design, coding, and iterative refinement of the algorithm aimed at instantiating the tool for eventual clinical usage.

**Participants:** **AR**, **EP**, **LT**, **LM.**

**Input:** Includes clinical data sets, domain knowledge from *EP*, existing algorithms/tools/models that can be adapted, and the *Project Plan Package* including (but not limited to) its *Project Plan, Software Specification, Risk Assessment, Validation Plan*.

**Output:** A containerized (e.g., docker/apptainer) version of the algorithm along with a fully drafted *Software Specification*, which includes items like well documented source code, hardware/software requirements for running the algorithm, and tests to be completed during 2.9 Validation Plan Execution Stage. The tool's user facing output/result format should be approved by *EP*, and in line with prior specifications.

**Timeline:** Whereas the complexity of the algorithm itself naturally influences the timeline—for instance, a simple IHC detection algorithm may take only a few weeks to develop, whereas a comprehensive image-based biomarker system could require month to years—this component is often the most *predictable*. Development efforts generally follow patterns from similar “research-use-only” or software development projects. By contrast, the factors discussed below in *Context and Considerations* are less constrained and more variable, often driving uncertainty and delays. Clarifying them early helps create a realistic, reliable project timeline.

**Context and considerations:** A key consideration is assessing data availability and allocating adequate time for data collection. Studies may require: (a) ethics approval, (b) retrospective curation of WSIs and associated clinical metadata, and (c) translation of this data into anonymized machine-readable formats suitable for tool development. As data volumes increase, the time needed—particularly for tasks requiring manual effort—is often underestimated.

Another key consideration is the choice of technology used to develop the algorithms. Some platforms may be less suitable for integration into high-throughput clinical workflows. For example, whereas MATLAB is well-suited for prototyping, Python is more widely accepted for production-grade development and integration. Selecting technologies that support scalability and compatibility with existing clinical systems and *IT* infrastructure from the outset is essential.

It is strongly recommended to avoid changing technologies between development and deployment, as both 2.4 Performance Evaluation Stage and 2.9 Validation Plan Execution Stage benefit significantly from using a consistent technology stack. Any changes at this point could invalidate the results of the 2.4 Performance Evaluation Stage and necessitate repeating the 2.8 Algorithm Hardening Stage.

**Worked HPyloriDet example:** Development began with *EP* annotating 20 HP positive WSIs (SVS or MRXS format), selected for their abundance of stained objects affording exposure to both clear true positives and potential false positives. To improve tool sensitivity, regions with ambiguous staining—where HP positivity was uncertain but would still warrant pathologist review—were labeled as positive. The model was developed in Python using the PyTorch deep-learning framework. After tissue detection was performed using thresholding on a 2.5× magnification black-and-white version of the WSI, ROI detection was built using stain deconvolution and applying a minimum DAB-stain intensity threshold. During training sensitivity, specificity, positive-predictive value (PPV), and negative-predictive value (NPV) were tracked, and errors manually reviewed, paying particular attention to false negatives. The pathologist-facing results display reached its final design, as shown in Fig. 2. For integration, DICOM support was implemented to ensure IMS compatibility: slides are downloaded from the IMS in DICOM format, annotations are transmitted through the IMS API, and image results are transferred via DICOM C-Store.

### 2.4 Performance evaluation stage

**Scope:** To evaluate the algorithm's performance on retrospective data in a way that informs whether it merits deployment and prospective validation.

**Participants:** **AR**, **EP.**

**Input:** An algorithm, as developed in 2.3 Development Stage, along with its associated *Project Plan Package* and *Validation Plan*, outlining key metrics and the corresponding datasets to compute them on.

**Output:** A scientific manuscript style document, termed a *Performance Report*, including sections for: Methods, Experimental Design, Experimental Results, Discussion, and Conclusion. Results should be presented both as aggregate summary statistics, and at the level of individual patients and/or items (e.g., patches), as inspecting metric distributions may offer deeper insights into potential failure modes. Evident failures of the algorithm should be noted in detail, as these will provide critical information for either: (a) refining the algorithm (return to 2.3 Development Stage, or address during 2.8 Algorithm Hardening Stage), (b) amending the *Risk Assessment* or *Software Specification*, and/or (c) inclusion into the 2.12 Standard Operating Procedure (SOP) Stage as documented edge cases for awareness of downstream tool users.

**Timeline:** For fully automatic algorithms with known available ground truths, the evaluation process can take only a few minutes, as the computational comparison of the algorithm output to the ground truth can be measured in an unaided way. On the other hand, it is often too expensive in terms of human labor to collect ground truth for use cases which have large numbers of objects (e.g., cell phenotyping), so a visual verification approach may be warranted, wherein the *EP* grades results on a semi-qualitative scale indicating {poor, modest, acceptable, ideal}, with requirements defined as a percentage of these categories. The challenge in this context is notably that this involves additional *EP* time, which can add delays to the overall process. Documentation timeline is similar to that of an equivalent peer-reviewed scientific manuscript.

**Context and considerations:** From our experience, there is often a large gap between the public research cohorts which algorithms are typically built with (e.g., Grand Challenge datasets,^40^ see Fig. 3) versus those with which the tool must operate on daily. Clinical data often involve greater complexity and heterogeneity, but typically maintains consistently high quality. As a result, to be more efficient in the evaluation, it is not uncommon to break the evaluation into increasingly larger steps. For example, a pilot study on a small subset of slides can and should be conducted to ensure that the results are as expected before beginning evaluation on the totality of the cohort. This enables a more rapid iteration, while also not exposing the algorithm to all the testing data, so that a confident assessment of the algorithm on held-out test data can still take place. Naturally, the closer this evaluation is to the real-world assessment (see 2.7 Validation Plan Generation Stage and 2.9 Validation Plan Execution Stage), particularly if the development and this performance evaluation only employ real-world data, the more efficient the overall validation process will be, as there will be fewer opportunities for unexpected situations to arise in later stages.

**Fig. 3:**
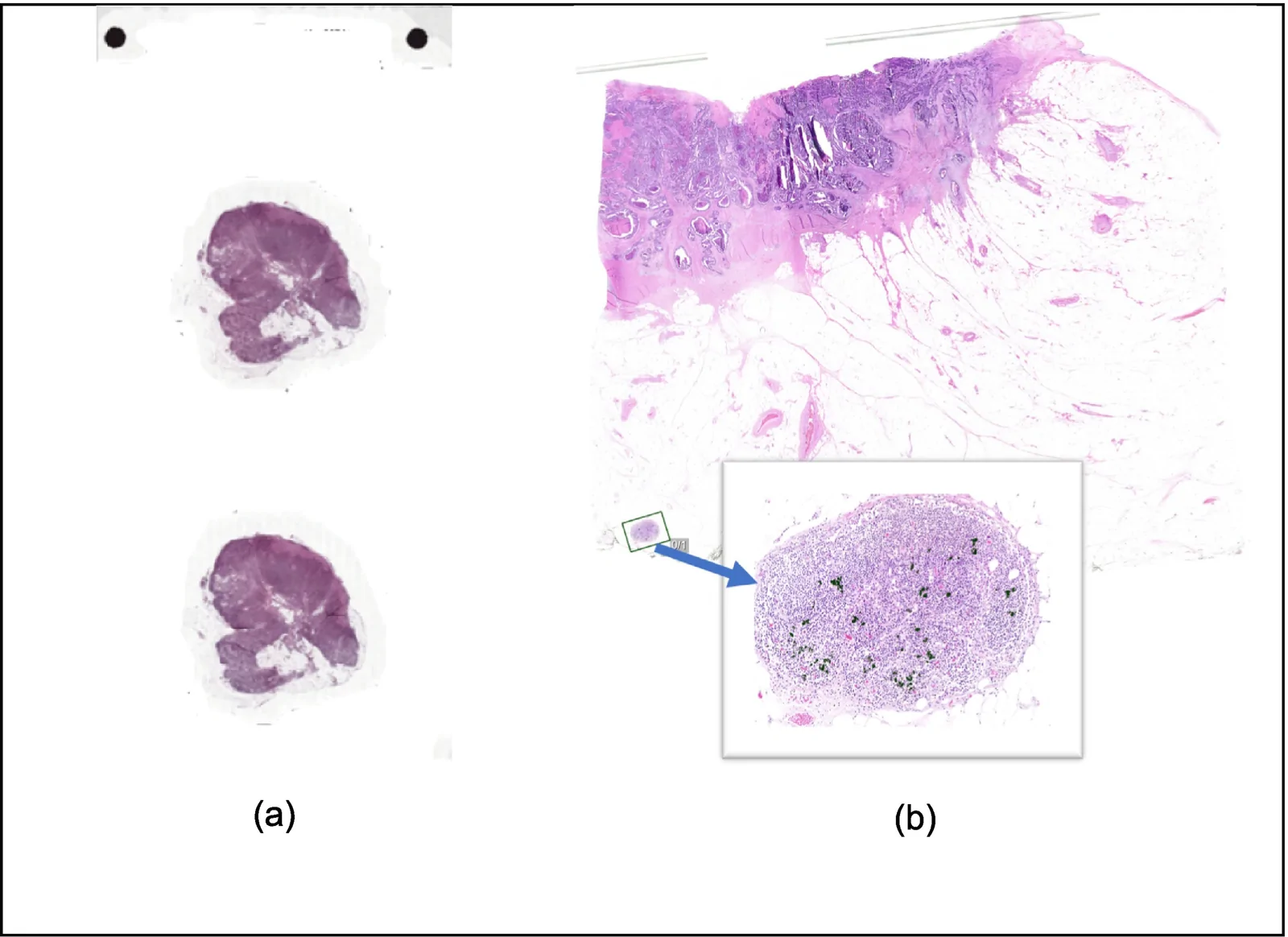
An example demonstrating the difference between: (a) a public research dataset for metastasis detection in lymph nodes, versus (b) a routine clinical slide, with the equivalent lymph node region highlighted in the bottom left box. When transitioning an algorithm developed using cohorts on the left to the real-world data on the right, it is likely that a performance gap will be exposed which will need to be addressed before the algorithm can be integrated into a tool meeting the needed performance specifications.

**Worked HPyloriDet example:** A *Performance Report* was generated for HPyloriDet, beginning with a summary of its functionality and representative examples of output within the IMS. The evaluation proceeded in three parts: (i) patch-level performance metrics (sensitivity, specificity, PPV, and NPV) were computed on the validation set of the 20 annotated slides (see 2.3 Development Stage), supplemented by visual inspection of false negatives and illustrative patch examples; (ii) a broader assessment was conducted on 132 routine histopathology slides collected over a 2-week period, with the primary aim of confirming correct identification of HP-positive cases, noting minor output issues (e.g., scattered false-positive patches) versus major issues (e.g., widespread false positives or predictions off tissue), and illustrating representative contact sheet outputs; and (iii) a preliminary estimate of time-to-diagnosis gain was derived using QuPath outside the IMS environment. The QuPath analysis suggested a ∼20% reduction in review time for negative slides; however, absolute review times differed substantially (∼2 min in QuPath vs. 20–30 s in IMS), likely due to: (a) the pathologist's greater familiarity with IMS and smoother navigation (e.g., 3Dconnexion “Spacemouse Pro” support), and (b) the QuPath-aided read being performed before inception of the contact sheet output format.

Several limitations were identified at this stage, including positive predictions occurring outside tissue regions (e.g., on artifacts), inability to ignore juxtapositioned control tissue when present on slides, and a relatively high rate of false positives—likely attributable to an initial training strategy focused on minimizing false negatives. Future development priorities included integrating quality control mechanisms, accommodating control tissue, and retraining the model to reduce false-positive rates. In hindsight, pre-collection of the 2-week slide cohort would have streamlined the assessment process.

### 2.5 Production deployment decision stage

**Scope:** Evaluate the algorithm's current performance, scope, and intended use case to determine its suitability for deployment.

**Participants:** **AR**, **DE**, **EP**, **LM**, **DL, IT (optional).**

**Input:** The *Performance Report* generated in the previous stage which summarizes the intended use of the algorithm, what validation was performed, on what data, the results of that evaluation in contrast to those expected and identified during the 2.1 Pre-Development Stage.

**Output:** A go/no-go decision by *DL* to proceed with the deployment and validation process. For traceability a *Decision Document* is created, being the signed version of the final *Performance Report* with any additional comments or notes which need to be propagated to subsequent stages.

**Timeline:** With appropriate documentation in place, a decision can often be reached in one or two meetings. That said, however, it is not uncommon that during the review process of *Input*, additional specificity or refinement is needed to inform the final decisions, and thus iterations of *Input* between the *Participants* to generate an approved *Decision Document* are common.

**Context and considerations:** The validation/accreditation process requires a dedicated effort to be completed, which stretches across many different departments, domains, and disciplines. As such, it is not “free” to validate/accredit algorithms, and only those that are likely to: (a) meet the required standards, (b) provide the expected added value, and (c) justify long-term support and maintenance should proceed with this process. It is also important to factor in that as one validates more algorithms of the same class, and better workflows and document templates are created, the easier the accreditation is likely to become, thus further increasing the number of algorithms which pass from the “research” into the “clinical” sphere.

**Worked HPyloriDet example:** HPyloriDet was approved to move into the Deployment Phase by *DL* upon review of the *Performance Report*.

### 2.6 Risk assessment stage

**Scope:** Analyze and assess all risks associated with deploying the tool into routine operation.

**Participants**: **AR**, **DE**, **EP**, **LT**, **QO.**

**Input:** The *Decision Document*, as well as established laboratory SOPs that connect to this tool. For example, most DP tools will be inserted into accredited DP workflows, which themselves contain a set of accreditation documents. Reviewing these to identify any interconnectivity that may pose a risk for the tool under consideration is suggested.^41^, ^42^, ^43^

**Output:** A formalized *Risk Assessment* document which aims to highlight the frequency, severity, and detectability of risks both before and after their respective mitigation strategies are employed.

**Timeline:** It is reasonable to expect this stage to take approximately 1 week, appreciating that the first time one completes it may require substantial time and effort. However, tools within the same class (e.g., diagnostic, prognostic, detection, counting) tend to share a core set of risk profiles, supporting reuse and refinement of templated risk tables. As a result, subsequent revisions and iterations are expected to proceed with increasing efficiency in a matter of hours, as familiar risks are systematically identified, scoped, and addressed.

**Context and considerations:** Assessing risk is one of the major components of the validation process, which as a reminder is focused on guaranteeing the quality, reliability, and safety of its processes and outcomes.

As *RP* and *LT* increasingly rely on deployed tools, it is crucial to consider the implications of tool failures. Any malfunction or unexpected behavior can significantly disrupt clinical workflows and potentially compromise patient care. Understanding the potential impacts of tool downtime or errors is therefore essential. Risk assessment must include scenarios, where the tool stops working as expected, examining both the consequences for clinical operations and patient outcomes, as well as how such failures can be rapidly detected through continuous monitoring and quality measurements. Contingency plans should be developed to address these scenarios, ensuring that alternative processes or manual interventions are in place to maintain continuity of care. Additionally, the risks extend beyond patient care to include workflow disruptions, cybersecurity threats, data leakages, and the ongoing training of pathologists to ensure they do not experience a loss of competence in interpreting results independently due to reliance on the tool.^7^

The insights gained from risk assessment directly inform the 2.7 Validation Plan Generation Stage. By identifying potential risks, the validation plan can incorporate specific tests and procedures to ensure that the mitigation strategies are effective in practice. This implies designing validation protocols that simulate real-world conditions and potential failure scenarios to verify the tool's reliability and robustness. For instance, if a risk is identified that the tool might misinterpret certain types of data, the validation plan should include extensive testing on these types to evaluate and improve the tool's performance under those conditions. Ensuring that risk mitigation approaches are thoroughly tested helps build confidence in the tool's readiness for routine use.

**Worked HPyloriDet example:** The primary risks associated with HPyloriDet were identified as: incorrect algorithmic HP detection, failure of the tool to execute, misinterpretation of results by the user, and potential impacts on overall system performance and workflow. Regarding risk of incorrect detection—where the tool may miss true-positive regions—a low frequency and detection risk was anticipated, with medium clinical gravity (see *Risk Assessment*). Mitigation strategies included a formal validation plan to assess detection performance, clearly communicated guidance to *RPs* regarding scenarios, where the output may not be reliable, and *explicit retention of the RP's final diagnostic authority*. The latter clearly signifying that HPyloriDet is not an autonomous tool, but a computer-aided diagnostic assay. HPyloriDet execution failure, although unlikely and of low impact, was mitigated by fallback to standard unaided slide review. To prevent misinterpretation—such as control tissue being mistaken for a patient's positive sample, or assuming high-confidence patches are always correct—training sessions and SOP documentation were defined, and further algorithmic improvements were planned to explicitly exclude control regions. Finally, to assess the potential impact on computational resources and workflow, HPyloriDet's resource requirements were benchmarked in a test environment before deployment. A dedicated high-performance server for DP tools was purchased, providing a scalable infrastructure for production. Testing and execution were carried out on the same system, enabling a more accurate risk assessment than would be possible using the previous desktop environment. Monitoring metrics were defined, aiming to detect systematic deviations from validated performance to ensure ongoing reliability (see 2.15 Monitoring Stage).

### 2.7 Validation plan generation stage

**Scope:** Define performance goals, expected criteria, and the methods to be used including number of samples, execution, and metrics.

**Participants:** **DE**, **LT**, **EP**, **QO.**

**Input:** In practice, all previous inputs should be present to ensure that the *Validation Plan* covers the entire scope needed for validation. Of particular importance is the *Risk Assessment* plan and *Software Specification*, as these will have identified concerns of varying levels of severity, that should be particularly addressed and evaluated during validation.

**Output:** A revised *Software Specification* and a *Validation Plan* (see *Appendix 3, 1. Planning Section*) that outlines the specific steps, methods, and metrics to be used in evaluating the tool. This includes defining the number and type of samples to be tested, execution plan, and performance metrics to be measured. The *Validation Plan* should specify objective acceptance criteria, such as accuracy, sensitivity, specificity, reliability, or time-gain that the tool must meet to be considered successful. Additionally, it should include procedures for analyzing data, recording results, and reporting findings. An ideal, well-structured, *Validation Plan* ensures that the validation process is systematic, transparent, and reproducible.

**Timeline:** Generation of the first *Validation Plan* is likely on the order of 100 h, with subsequent ones on the order of 10 h. Similar to the *Risk Assessment*, the first *Validation Plan* may take longer as the requirements should be iteratively fleshed out with the *QO* and potentially the *AC*, but once in place, generation for subsequent tools of the same class should be increasingly efficient as core requirements are not likely to change significantly.

**Context and considerations:** This stage involves understanding the purpose and scope of the validation process and ensuring it aligns with clinical and regulatory requirements. The primary purpose is to define a *Validation Plan,* which enables the verification of the tool performing as intended and meets predefined performance goals. This includes confirming that improvements in any subsequent versions of an established tool enhance its functionality as compared to the previous version.

Important considerations include the clinical relevance of validation metrics, representativeness of the sample data, and feasibility of validation procedures within clinical settings. It is important to anticipate potential challenges, such as variability in sample quality or integration issues with existing systems, and include strategies in the *Validation Plan* to ensure they are effectively addressed. For example, the 2.6 Risk Assessment Stage helps determine the types and characteristics of cases, especially complex or edge cases, needed during this validation. The pathologist/end-ruser's experiences using the tool in their routine practice remains crucial, thus planned dedicated surveys documenting their acceptance and approval is warranted.

Time spent during earlier stages reflecting on validation requirements should now yield benefits, as the *Validation Plan* may already be largely in place due to those efforts.

**Worked HPyloriDet example:** Building on the successful performance evaluation (see 2.4 Performance Evaluation Stage), the following acceptance criteria were established for promotion of HPyloriDet to production:

1. *Detection and removal of positive control samples placed adjacent to patient tissue*: When not properly identified, these highly positive regions overwhelm patient-specific results, causing a 5%–10% slide-level false-positive rate. A key task identified for 2.8 Algorithm Hardening Stage, was to accurately exclude this control tissue computationally. The acceptance threshold was set so that HPyloriDet must correctly handle more slides than simply ignoring all slides containing control tissue (which would be the default fallback). This functionality was evaluated in 2.9 Validation Plan Execution Stage.
2. *Patch-level sensitivity and specificity*: For acceptance, patch-level sensitivity and specificity was set to at least match those from the 2.4 Performance Evaluation Stage. Whereas these metrics are critical for rapidly computationally assessing HPyloriDet, the primary focus remained on slide-level accuracy, i.e., correctly identifying slide HP status using the top 20 predicted regions. As such, patch-level metrics acted as a computational surrogate appreciating that they do not fully capture clinical relevance. Manual review of false negatives patches was also required, and false negatives were considered acceptable if they occurred in patches with ambiguous staining, but were not acceptable in any patch showing clearly visible HP. Validation used the original evaluation patches along with any additional patches collected during the hardening phase.
3. *False-negative rate*: As a sensitive screening tool, HPyloriDet was required to identify all positive slides The acceptance criterion mandated that at least one of the top 20 predicted regions on every positive slide must contain HP. This requirement was evaluated retrospectively using the 132 sequential slides from the 2.4 Performance Evaluation Stage, and prospectively during the survey period (see *List Item 5***,** below). Slides exhibiting known failure modes—identifiable by pathologists within the IMS—were excluded. These included errors in tissue control handling, major quality control failures such as all top 20 predictions highlighting artifacts, incorrectly identified tissue regions, predictions falsely positive on red blood cells, blurry slides, and slides containing unusually large tissue samples such as surgical specimens. These situations were appropriately documented in the 2.6 Risk Assessment Stage and 2.12 SOP Stage.
4. *Time saving*: A reduction in time to diagnosis serves as the primary measure of clinical benefit. For acceptance, HPyloriDet had to demonstrate a statistically significant improvement within the IMS, as used by pathologists, in average time to diagnosis (*p* < 0.05) compared to routine slide examination without it. For rigor, the validation targeted matched numbers of positive and negative slides, with all slides containing comparable amounts of tissue. Slides meeting exclusion criteria outlined in *Item 3* were omitted from analysis.
5. *Pathologist survey*: A mandatory survey would be conducted to assess *RPs* confidence in the tool's outputs. For acceptance, the average confidence rating must be at least 2.7 out of 3,^44^, ^45^ with high = 3 points, medium = 2 points, or low = 1 point. Slides excluded under the criteria outlined in *Item 3* were not considered in this evaluation.
6. *Average analysis time*: HPyloriDet must process slides quickly enough to ensure result availability when *RPs* review cases, particularly because IHC-stained slides are batch scanned in the late afternoon. Acceptance requires that average total analysis times remain below 5 min, including downloading, analyzing, and uploading results for each slide.
7. *Repeatability and reproducibility*: HPyloriDet had to demonstrate consistent performance, with reanalysis of the same slide yielding identical top 20 patch scores to 2 decimals. Furthermore, analyses of the same slide performed on the two different scanner models used in our clinical workflow must yield the same diagnostic conclusions.

Based on the *Software Specification*, the following criteria were identified for validation:

1. *Unit tests*: Passing unit tests for key functions.
2. *Integration tests*: Before deployment, HPyloriDet must complete successfully in the IMS test environment on one positive and one negative slide per scanner, with results displayed as expected.
3. *Resource usage*: The maximum RAM and VRAM usage must remain below 10 and 5 GB, respectively.
4. *Concurrent usage/reliability*: Using two concurrent HPyloriDet instances, all WSIs must be processed so that results are available for pathologists to begin case review according to their regular rhythm of clinical workflow.

### 2.8 Algorithm hardening stage

**Scope:** Transforming a research-grade *algorithm* into a robust *tool* suitable for clinical use.

**Participants:** **AR**, **DE**, **IT.**

**Input:** A research grade algorithm along with the *Validation Plan* and *Risk Assessment.*

**Output:** A fully hardened tool integrated and working in the clinical environment, for execution in 2.9 Validation Plan Execution Stage. A revised *Software Specification* incorporating results from execution of the *Software Verification Plan*. The code should be well documented and have undergone code review to ensure: (a) adherence to best practices and internal coding guidelines (e.g., SDK usage), and (b) understandability by *LT*.

**Timeline:** Here again, the advantage of having precise tool specifications becomes evident. If the tool was developed with the required standards in mind, this phase can be completed quickly and with minimal effort. However, if those standards were not adequately addressed early on, substantial rework may be needed to achieve the necessary performance, throughput, and integration. Consequently, this phase can range from just a few hours to several months, depending on the alignment between the prior 2.3 Development Stage and 2.7 Validation Plan Generation Stage stages.

**Context and considerations:** The transition from a *research algorithm* to a *clinically deployable tool* involves: (a) refining the approach to meet strict performance and regulatory standards, (b) integrating it seamlessly into the clinical ecosystem, and (c) preparing it for rigorous validation according to the *Validation Plan*. During this hardening process, *DE* and *AR* collaborate closely: *DE* is responsible for adapting the algorithm to the healthcare facility's technical infrastructure, ensuring compatibility with existing electronic health records (EHRs) and associated systems such as lab information systems (LISs), and IMS. Meanwhile, *AR* provides ongoing support and adjustments based on all prior feedback. Together, they optimize and test the algorithm's performance to handle real-world clinical data with high accuracy and reliability, while respecting any IRB requirements (e.g., AR may potentially not have access to clinical data). This may include fine-tuning parameters by specimen type (e.g., biopsies vs. resections), improving computational efficiency, and ensuring the tool can process large data volumes rapidly without compromising accuracy.

Security and compliance are critical aspects of algorithm hardening. *DE* must ensure the algorithm complies with data privacy regulations and cybersecurity best practices. This often involves collaborating with *IT* and data security officers to implement measures such as data encryption, secure transmission protocols, and safeguards that prevent unauthorized access to sensitive patient information.

Lastly, robust error handling and monitoring are essential, in particular to mitigate risks identified in *Risk Assessment*. The system should include mechanisms for detecting and managing crashes or failures, with comprehensive error logging that captures sufficiently detailed diagnostic information to enable debugging. Logs should be securely stored and accessible for troubleshooting and auditing purposes. Error reporting should be automated, with alerts sent to relevant *LT*/*DE* for timely intervention. Integrating the tool into a centralized monitoring dashboard enables real-time tracking of errors, performance metrics, and key performance indicators across all deployed tools. This centralized approach facilitates rapid identification of issues, trend analysis, and supports continuous improvement. Additionally, automated fail-safe procedures should be implemented to ensure that, in the event of a critical failure, disruption is contained within the failing component. These mechanisms should prevent cascading failures by isolating faults, enabling a controlled and limited shutdown that minimizes impact on other algorithms and preserves overall clinical operations.

Importantly, this is the last time that the tool should be refined and adjusted, as once it is validated, any subsequent modifications will require re-testing, reporting, and documentation. As a result, carefully reviewing the *Validation Plan* to ensure that the algorithm is in line with expectations is critical.

**Worked HPyloriDet example:** During the algorithm hardening phase, a beta version of HPyloriDet was deployed in routine clinical practice. It was evaluated through multiple rounds of *RP* surveys with clear indication that it was not yet validated for use as a clinical aid (i.e., Research Use Only). To facilitate deployment, HPyloriDet was containerized (docker) and hosted on a dedicated high-performance computing server, with two additional containers for: (a) logging and managing daily slide monitoring, and (b) supporting the HP feedback mechanism (see Fig. 4).

**Fig. 4:**
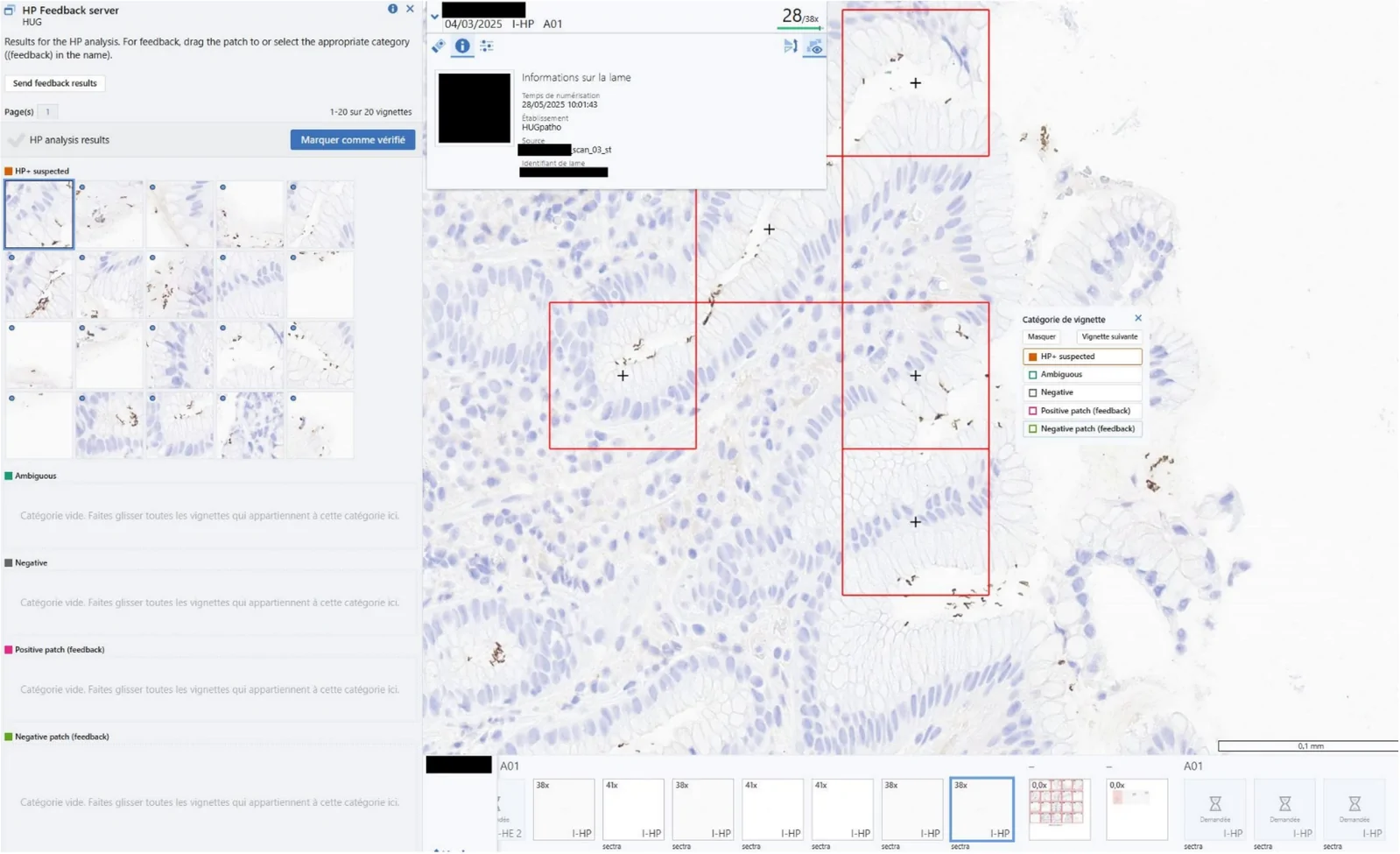
Gallery View within the Sectra viewer, which enables users to review algorithm predictions and provide corrective feedback. Predicted patches are organized into categories: “HP+ suspected” (high-confidence positives), “Ambiguous” (low-confidence positives), and “Negative.” Two additional categories, “Positive patch (feedback)” and “Negative patch (feedback),” allow users to reassign labels either by dragging patches into the correct group or by selecting the appropriate category. Once feedback has been applied, updated labels can be stored by clicking the “Send Feedback Results” button.

A significant improvement involved enhanced control handling: when control tissue was detected, it was automatically excluded from the analysis, and the omitted regions were clearly highlighted on the “Regions analyzed Mask” for pathologists' reference (see Fig. 2). Additionally, an in-IMS feedback mechanism was implemented (see Fig. 4), allowing pathologists to flag patches as correctly or incorrectly classified directly within the viewer, enabling real-time feedback during routine use.

Quality control was another focus area. After the initial survey yielded a confidence score of 2.5—below the target of 2.7—largely due to numerous false positives, HistoQC^4^ was integrated to filter out artifacts outside of tissue areas. This integration helped reduce slide-level artifacts and improve overall tool accuracy.

Despite improvements, the second survey round only raised pathologist confidence score to 2.6, still short of the target. In response, the model was retrained using patches flagged by pathologists during routine practice, as well as selected HP analysis output patches saved during regular use.

### 2.9 Validation plan execution stage

**Scope:** Conduct validation tests using both retrospective (previously analyzed cases) and prospective (ongoing case analyses alongside the validated method) approaches.

**Participants:** **LT**, **QO**, **EP**, **RP.**

**Input:** Completed *Validation Plan* Planning Section.

**Output:** Metrics and values, including *RP* surveys, needed for the completion of the Report Section of the *Validation Plan*.

**Timeline:** The duration may vary significantly based on the associated *Validation Plan* and ability to meet the acceptance criteria. A primary factor contributing to this variability is the minimum number of slides required for evaluation, which directly ties the completion time to the case load managed by the specific tool.

**Context and considerations:** During the execution of the *Validation Plan*, it is crucial to closely monitor and document any deviations from expected outcomes, such as unexpected false positives or false negatives that may not have been anticipated during the earlier 2.6 Risk Assessment Stage or 2.8 Algorithm Hardening Stage phases.

Identifying these deviations promptly allows for this process to evolve into an iterative cycle, where new findings—especially rare or edge cases that were too infrequent to be randomly sampled in retrospective evaluations—may prompt a return to earlier stages, including 2.6 Risk Assessment Stage, 2.7 Validation Plan Generation Stage, and 2.8 Algorithm Hardening Stage. Rather than viewing these discoveries as failures, it is important to recognize them as valuable opportunities for continuous improvement. Such iterative refinement ensures that the tool ultimately deployed maintains safety, reliability, and robustness, which support the delivery of the highest quality patient care.

Recording analyzed cases in this stage facilitates long-term traceability and enables re-evaluation as the tool evolves.

**Worked HPyloriDet example:** During evaluation, insufficient confidence levels on the pathologist survey were addressed by reverting back to the 2.8 Algorithm Hardening Stage to: (a) integrate HistoQC and (b) perform additional model retraining based on annotations provided during routine clinical usage. Once both were implemented, this stage was re-executed, and results curated.

### 2.10 Validation plan compilation stage

**Scope:** Completion of the *Software Specification* and *Validation Plan*, incorporating final statistics and conclusion.

**Participants:** **LT, QO.**

**Input:** Test results from the previous stage and the associated *Validation Plan*.

**Output:** A completed: (a) *Software Specification* and (b) “Report” Section of the *Validation Plan*, converting it from a *Validation Plan* into a *Validation Report.*

**Timeline:** If all data are present, computing the final statistics and writing the report typically takes less than 10 h.

**Context and considerations:** Summarizing the results within the institutionally approved templates typically requires noting any discrepancies identified during the testing phase. These should be meticulously documented, analyzed, and addressed to both refine the tool, if needed, but as well be taken into consideration when developing 2.12 SOP Stage to help mitigate any risks associated with its clinical application.

As seen in *Validation Report (Section 2 Item 7)*, the Conclusion Section essentially states if the tool met the required operating characteristics and should be considered for deployment. If this process fails, i.e., specific performance criteria are not met, one needs to determine if it is a catastrophic failure, requiring reverting back to 2.3 Development Stage, or if the failure is due to relatively minor points that can be addressed by proceeding with additional 2.8 Algorithm Hardening Stage**;** in either case, a revision of the *Validation Plan* and/or the *Software Specification* may be needed.

Notably, at this advanced stage, either outcome is considered costly and therefore should aim to be avoided whenever possible. Such situations may reflect insufficient clarification of the project scope or an incomplete assessment of anticipated risks. In particular, when completing this phase using an internally developed tool, unforeseen issues should be rare—any potential challenges should have been identified and addressed during earlier stages of development and planning.

**Worked HPyloriDet example:** The results for the validation were the following:

*1. Control detection rate**:*** Of 103 validation slides (9 with controls), HPyloriDet correctly identified 5 controls, retaining 99 slides for analysis versus 94 without control handling, exceeding the stated baseline tolerance (see 2.7 Validation Plan Generation Stage List item 1). Notably, during the survey period, all 172 slides had the control handling performed correctly, indicating that the effective rate in practice was higher than observed in the validation step. The primary source of residual error was attributable to coverslip artifacts.

*2. Patch-level sensitivity and specificity:* An additional 630 patches were added to the original validation dataset, bringing the total to 5316 patches with 549 being positive. The re-trained model retained a sensitivity of 93%, while improving specificity from 88 to 94%. Importantly, all patch-level false negatives occurred in regions with ambiguous staining—no patches with clearly visible HP were missed.

*3. False-negative rate (slide-level)*: Across both original validation slides and cases evaluated during the survey period, no positive slides were missed, yielding a 0% false-negative rate. One slide was excluded from analysis during the survey due to the predefined documented criteria.

*4. Time to diagnosis*: An experiment comparing aided versus unaided diagnosis was conducted on 25 slides per condition (with 9 positive cases in each). The tissue burden was matched across groups to ensure comparability (3 low, 9 medium, and 4 high tissue volume slides in the negative set). Tool-assisted review led to an average 26% reduction in time to diagnosis (26.68 s vs. 36.21 s, *p* = 0.02), meeting acceptance criteria.

*5. Pathologist confidence survey*: Over a 2-week survey period, 80 cases and 172 slides (33 positive) were reviewed. One case was excluded due to slide debris exceeding QC tolerances. The average confidence score reported by *RPs* was 2.93 out of 3, exceeding the required threshold of 2.7. Only one case had a low confidence rating due to black ink generating excessive false positives. Medium confidence was reported in four additional cases: two involving predictions outside tissue, one due to over-prediction on red blood cells, and one positive case with only a small amount of HP, where HP regions were correctly displayed in the top 20 patches but the pathologist noted that a relevant patch was not included.

6. *Average analysis time*: HPyloriDet processed slides in an average of 1 min and 50 s, well below the 5-min threshold required for clinical viability.

*7. Repeatability and reproducibility:* The tool produced identical results when run multiple times on the same slide and generated comparable outputs when analyzing the same slide scanned on different scanners, confirming its consistency across runs and hardware variations.

All *Software Specification* requirements were also met, including: (a) unit tests, (b) integration tests, (c) resource usage limits, and (d) concurrent usage/reliability tests as described in 2.7 Validation Plan Generation Stage.

### 2.11 Sign off for production stage

**Scope:** The final validation report is reviewed and approved.

**Participants:** **QO**, **LM**, **DL.**

**Input:** Completed *Validation Report* and *Software Specification*.

**Output:** Go/no-go decision after reviewing *Validation Report* and *Software Specification*.

**Timeline:** This stage can be completed in as little as a few hours, but will depend highly on the time required for each signing official to review the documentation, provide feedback, and resolve any outstanding questions or concerns.

**Context and considerations:** The main considerations at this step are a thorough review by the responsible parties to ensure the documents and tests have been completed as needed for promotion of the tool to routine clinical usage.

**Worked HPyloriDet example:** The tool was approved for deployment in production without incident.

### 2.12 Standard operating procedure (SOP) stage

**Scope:** Drafting the SOP describing the usage of this new tool.

**Participants:** **DE**, **LT**, **QO**, **EP.**

**Input:** *Validation Report* along with feedback from the various stakeholders who have experience in using the tool.

**Output:** A versioned SOP uploaded to the institutional document management system.

**Timeline:** Producing a complete first version of the SOP typically takes around 1 week, and may be faster for subsequent tools as some sections are reusable. However, multiple iterations are often part of the broader revision process, as feedback from stakeholders is gradually integrated to ensure the SOP is clear, comprehensive, and aligned with clinical expectations. Certain sections—such as troubleshooting—may continue to expand over time, with subsequent revisions issued as new issues arise during routine use. In this sense, this is a “living” document, which implies this stage does not terminate, but instead becomes less frequent overtime as the tool matures.

**Context and considerations:** The development of SOPs is critical to the correct usage of the tool. Items that are typically included are listed in *Appendix 5 - Standard Operating Procedure Template*. These SOPs cover a few core concepts: (a) when and what the tool should be applied to, (b) who should use the tool, (c) how they should use it, (d) installation, (e) maintenance and backup, and (f) troubleshooting. When drafting the SOP, it is worthwhile to think about who will be reading it and when. For example, most likely, experienced users or developers are not regularly reviewing the documentation. It is more likely that the tool has unexpectedly broken, and *IT*/*LT* are looking for ways to repair/restart it, or new people (e.g., *RP*) are onboarded and aiming to learn how to use the tool safely. Considering these points will yield an SOP, which is not only needed for validation but also improves usage by its users.

**Worked HPyloriDet example:** SOPs were divided into two main components, tailored respectively for *RPs* and *LTs*:

*For RPs:* The SOP outlines both access and use of HPyloriDet within their clinical workflow. This includes stating that: (a) to view results, the *RP* must have access to the IMS and be working on a case involving an HP-IHC slide and (b) results are integrated directly into the IMS environment, appearing as DICOM images positioned next to the analyzed slide and as overlaid annotations on the slide itself. Importantly, *RP* is informed that the analysis is launched automatically in the background when a slide is received by the IMS, and results are typically available within 5 min after completion of the slide scanning process.

The SOP also includes detailed guidance on result interpretation. This section explains the presentation format of the outputs, highlights illustrative examples for clarity, and outlines known tool limitations (e.g., exclusion criteria described in 2.7 Validation Plan Generation Stage). Finally, it provides instructions for submitting feedback via the integrated viewer, in line with the tool validation protocol described in 2.8 Algorithm Hardening Stage.

*For LTs:* The SOP focuses on the installation, operation, and ongoing maintenance of the system. It specifies the location of the codebase and documentation, lists required supplementary files with corresponding storage paths, and provides instructions for containerization—detailing how to build and deploy the analysis container. To prevent data duplication or configuration drift, certain technical elements may instead opt to be cross-referenced from other systems, such as README files in version control.

Maintenance procedures include a description of potential errors, how to identify and address them, and how to restart the container when necessary. To ensure traceability, the SOP also defines the storage structure and content of log files, outlining what is retained and where it can be accessed for audit or debugging purposes.

### 2.13 Team training stage

**Scope:** Provide training for the relevant individuals identified in the SOP.

**Participants:** **DE**, **LT**, **RP**, **QO.**

**Input:** Access to a running instance of the tool, along with the SOP.

**Output:** Record training as completed for the individuals in the training register, which marks them as competent to use it.

**Timeline:** Depending on the sophistication of the tool, the training can typically be completed in a few hours.

**Context and considerations:** As stated by current standards, the lab needs to identify the competence requirements for each tool to ensure that users are able to use the tool correctly.^46^ This training is typically done via interactive sessions, where users are introduced to the tool, its capabilities, and its integration into their workflow. These sessions can be led by *DE* or *LT* who can provide in-depth explanations and answer questions. Practical sessions then follow where users can interact with the tool in a controlled environment, using intentionally curated sample data to simulate real-world scenarios. This helps users gain confidence and familiarity with the tool.

Regularly scheduled sessions to update users on newly validated version of the tool may be required to ensure their skills remain current. Other approaches, such as online courses that cover various aspects of the algorithm, including theoretical background, practical use, and case studies, or live/recorded webinars may be used to provide supplemental information about the tool to increase familiarity if needed or desired.

The discretion to mark a user as competent is given to the individual conducting the training session, who may implement quizzes or assessments to evaluate understanding. In our experience, the SOP is explained, questions addressed, and the trainee instructed to complete the steps mentioned in the SOP on their own once, with the output verified as correct.

**Worked HPyloriDet example:** Similar to the SOP above, two dedicated training sessions were developed to ensure proper understanding by *RPs* and *LTs*.

For *RPs*, training included a comprehensive walkthrough of the SOPs and live demonstrations within the IMS. These sessions covered all essential components of tool usage, including how to access and interpret results, recommended practices, known limitations (such as exclusion cases outlined in 2.7 Validation Plan Generation Stage), and how to submit patch-level feedback directly through the system. Upon completion, *RPs* signed a confirmation form to document their participation. In addition, HPyloriDet training was incorporated into the standard IMS onboarding protocol for all new *RPs*.

For *LTs*, the session covered a SOP walkthrough and additional technical aspects of deployment and maintenance. These included detailed instructions on installation and configuration—covering code location, necessary files, documentation access, and container build and run procedures. The training also addressed maintenance and troubleshooting, offering guidance on handling common errors and restarting the container when needed. Finally, participants were taught how to manage records and maintain traceability through the automated structured logging systems. Like the *RP* training, completion is confirmed with a signed form.

### 2.14 Module ready for clinical production stage

**Scope:** Deployment to production checklist is filled, reviewed, and approved.

**Participants:** **LT**, **LM**, **QO.**

**Input:** All previous documentation.

**Output:** A signed *Appendix 4 - Production Deployment Checklist.*

**Timeline:** Execution time lies in the range of a few hours, as items should have in fact been addressed in previous phases. It is suggested to review this document from the onset of *Validation Phase* to ensure needed milestones are successfully reached along the way.

**Context and considerations:** A final check to ensure that all the needed documentation, training, and tests have been completed and put in place. Once signed, the tool is officially available for usage by all those competent to use it. As such, the checklist should contain all steps that should be verified, such as: (a) placement of the SOP into an accessible document repository, (b) availability of the computing resources, and (c) tool now enabled for daily use.

It is important to note that at this stage, although the tool has been validated as an LDT, it is *not yet accredited*. Upon DL's request, necessary documentation can be submitted by QO to initiate a request to extend existing accreditation to include this new tool. During the next scheduled audit visit—which typically occurs every 2 years—the tool will be reviewed onsite by the auditors (e.g., SAS). Based on their evaluation, accredited status may be granted. If accreditation is not awarded, necessary adjustments must be made to ensure that all regulatory requirements are fully met before resubmitting for approval.

**Worked HPyloriDet example:** All documents were approved, and HPyloriDet was promoted to production.

### 2.15 Monitoring stage

**Scope:** Monitoring of tool performance over time to ensure constancy in performance and functioning.

**Participants:** **LT**, **RP**, **QO**, **AC.**

**Input:** At a specified interval, a subset of cases reasonably covering the expected gamut of presentations, re-read unaided by a qualified user and compared against the tool's output.

**Output:** A *Monitoring Report* detailing the tests performed, slides used, and noting any variations from expected performance metrics identified in the *Validation Report*. This can essentially be viewed as a subset of the *Validation Report*, which focuses on repeating and confirming performance metrics. Additionally, the report identifies the personnel who conducted the tests, along with any anomalies observed, and any recommendations for algorithm refinement based on the findings.

**Timeline:** Monitoring occurs as long as the tool is in use, with reporting requirements at identified time intervals.

**Context and considerations:** The tool should have an established procedure for monitoring the validity of the results it produces. This implies recording results in such a way that trends and shifts are detectable and, where practicable, statistical techniques can be applied to review the results and identify sub-optimal behavior or outliers.

There are two types of monitoring which can take place, Internal Quality Control control (IQC) or External Quality Assessment (EQA), with some tools taking advantage of both approaches. A more complete discussion is available in “Ensuring the validity of examination results” of the ISO standard,^24^ but the concepts are briefly elucidated here.

For prospective IQC, a subset of slides is re-evaluated at a fixed time (e.g., quarterly) to ensure concordance with the performance previously demonstrated during the *Validation Plan*. Any discrepancies between the original and current performance mechanisms trigger a review of the tool or related processes to maintain its accuracy and reliability. EQA sees the results of the tool compared with results obtained by other labs via, e.g., the exchange of WSI, or third parties provided references standard samples for analysis. For accredited tools, when EQA is not available, period IQC is required.

To ensure ongoing reliability and safety, a formal mechanism for user feedback should be established. This enables early detection of unexpected shifts in tool performance, which may arise from sources such as stain variability, batch effects, or other latent variables (e.g., subtle changes in tissue thickness^47^) that can evolve during routine laboratory operation. User-reported concerns—particularly when aggregated—can serve as valuable early warning signals of tool degradation or emerging edge cases not previously identified.

In parallel, due to the computational nature of these tools, automated monitoring systems can be implemented to perform real-time statistical surveillance of performance metrics. These systems can apply well-established quality control rules—such as Westgard^48^, ^49^ or Nelson^50^, ^51^ rules—to detect statistically significant deviations from expected behavior. When triggered, such alerts can prompt immediate review or intervention, minimizing the risk of performance drift going unnoticed.

Together, these user- and algorithm-driven monitoring approaches form a robust feedback and quality assurance loop, ensuring the tool remains safe, accurate, and clinically valuable throughout its operational lifetime. Ultimately, however, it is important to recognize that a properly deployed algorithm—being deterministic and pinned to fixed software versions—should not drift on its own. Exiting from “in-control” performance typically indicates that an external factor in the surrounding system (e.g., staining, scanning, or data handling) has shifted, rather than the tool itself. In such cases, the priority should be to bring the input or upstream process back under control. Any algorithmic adjustments should be undertaken with great caution, as they may cross a threshold that effectively creates a new version of the tool, thereby invalidating prior validation and necessitating revalidation, starting from 2.8 Algorithm Hardening Stage. This process can be further complicated if significant time has passed since the original development, as the necessary expertise, development context, or training data may no longer be readily available.

**Worked HPyloriDet example:** Globally, the DP workflow at HUG undergoes periodic NEQAS quality evaluation (an EQA), which ensures the quality of slide cutting, staining, and digitization.^52^, ^53^ NEQAS requires both physical and digital slides for comparison; however, the DP module is currently available only for HE slides. To ensure the continued reliability of the HPyloriDet tool, in particular, both manual and automated review processes were implemented during deployment. Manual review was and will be conducted quarterly by selecting four representative slides per scanner model to verify that the tool produced consistent outputs across devices and maintained diagnostic concordance. This periodic check helped validate that hardware variability did not impact tool performance, and also occurs when, e.g., a scanner software update occurs.

In parallel, automated monitoring was implemented to continuously detect performance drift that might not be identified through manual checks alone. On the input side, HistoQC outputs are continuously monitored to detect shifts in slide quality, such as scanning artifacts or staining anomalies, which could impact tool reliability. On the output side, the distribution of prediction scores is tracked separately for slides predicted as positive and negative, along with the overall ratio of predicted positive to negative cases. This allowed the identification of gradual shifts in scoring behavior, which could suggest emerging issues with the model or upstream processing.

## 3 Discussion

Based on practical experiences in implementing a full DP workflow at HUG, this manuscript outlines essential steps for developing, validating and optionally accrediting a clinically deployable DP tool under the framework of a LDT. In addition to describing a generalizable pathway from research to clinical integration, we provide supplemental materials and templates for the core documentation needed throughout this process. Our discussion is further grounded in practical experience, drawing on the end-to-end development and validation of HPyloriDet, an in-house computer-aided detection (CAD) tool for identifying HP in gastric biopsies.

A clear understanding of the full workflow—from design through deployment—is essential for the successful development and validation/accreditation of clinical algorithms. By anticipating regulatory and operational requirements early, developers can integrate necessary elements organically, reducing the risk of major rework later in the process. This proactive, holistic approach also supports better coordination and feedback across multidisciplinary teams, enabling clinical, technical, and regulatory stakeholders to contribute meaningfully from the outset. In turn, this fosters transparency, alignment, and efficient decision-making. Ultimately, embedding validation considerations throughout the development cycle helps ensure a smoother, more robust pathway to clinical integration.

One important aspect not explored in depth here is the associated financial cost—particularly when partnering with third-party tool providers. It is essential to ensure that their business models align with the budgetary constraints of pathology services. Whereas a tool may demonstrate clinical utility, its implementation can lead to significant ongoing expenses, both anticipated and unforeseen. In addition to the considerable effort required for validation, integration often demands substantial technical resources, which can lead to hidden costs (e.g., custom integration fees, in particular with/for/to legacy systems). Institutions may also experience sticker shock post-integration when faced with recurring licensing fees or support costs. As such, it is critical to fully understand contractual obligations, billing structures, and the potential for reimbursement before committing to the development, deployment, and validation of any tool.

Regardless of the origin of the tool, be it in-house or via a third-party, both development and validation remain inherently iterative processes—particularly in the still-maturing field of clinical DP. These efforts should be viewed as scientific endeavors aimed at discovering and applying knowledge to real-world clinical systems in a rigorous and reproducible manner. Given the inherent complexity of biology and the variability encountered in routine practice, it is important to approach this work with humility and an understanding that the first iteration is unlikely to be perfect. Our own experience, shared here through the lens of a relatively simple diagnostic algorithm, reflects the culmination of more than 3 years of work by a mid-sized multidisciplinary team. However, the lessons learned through this effort have already significantly enhanced our capacity to develop and validate subsequent tools, several of which are currently under way within our institution.

One of the key lessons learned was the value of early technical prototyping to reduce uncertainty and manage risk. Whereas the initial time estimates for developing and validating the algorithm were fairly accurate—taking approximately 3–4 months in total—the integration phase, particularly with the IMS, proved significantly more time-consuming than anticipated. Much of this delay stemmed from the fact that it was our first attempt at transmitting DICOM images in this manner, revealing unexpected technical hurdles. In retrospect, having an operational prototype of that integration earlier in the process would likely have accelerated development. Since model training and platform integration can proceed in parallel to some extent, earlier prototyping could have allowed for more efficient allocation of resources and a reduction in overall development time. Relatedly, we recognized the value of developing a site-specific SDK to centralize and standardize common functions such as slide downloading, result uploading, and logging. Without such a framework, even minor updates become increasingly burdensome, as having a multitude of differentially implemented tools creates an unsustainable maintenance load for *DEs* supporting a growing collection of tools.

A key aspect of a long-term deployment is not only software maintenance but also monitoring for performance drift, as is required for all tools used in an accredited setting. Drift is primarily driven by changes in upstream processes; in accredited environments, these changes are ideally already subject to formal change control procedures. In practice, modifications such as installation of a new staining instrument, adjustment of staining protocols, replacement of reagents, or upgrades to scanner hardware or firmware are treated as controlled events that trigger re-validation of affected downstream components. In the context of computational pathology tools, this includes explicit consideration of the algorithm as a dependent consumer of slide-derived inputs, and therefore part of the validation cascade.

However, the extent of re-validation should be proportional to the magnitude of the upstream change. For example, introduction of a new scanner model is generally considered a “major change” due to known batch effects and documented sensitivity of deep-learning systems to domain shift, and would therefore warrant a comprehensive re-validation procedure. In contrast, replacement or upgrade within the same scanner model may be addressed through a more limited equivalence assessment, such as quantitative comparison of color stability (e.g., ΔE metrics between scans of the same reference slide) and verification that downstream algorithm outputs remain stable within predefined tolerances.

More generally, a practical and scalable strategy for drift detection is to exploit paired workflows when feasible: a subset of slides can be processed on both a validated reference system and the updated system, and concordance of algorithm outputs can be assessed at the level of the final diagnostic or decision-support output. When equivalence is demonstrated, the new configuration can be progressively promoted to routine use with reduced operational burden. In addition, and as discussed in 2.15 Monitoring Stage, continuous performance monitoring in routine workflows (e.g., tracking confidence distributions, output stability, and case-mix shifts) can help identify more subtle forms of drift that may not be immediately attributable to a single upstream change, thereby supporting early detection and timely re-validation.

That said, a key limitation in providing fixed benchmark ranges for (re-)validation metrics is that clinically meaningful thresholds are inherently use-case dependent and cannot be generalized across applications. Performance requirements are driven by the intended clinical role, risk profile, and downstream consequences of algorithm failure. For example, screening tools such as metastasis detection typically prioritize sensitivity to minimize false negatives, whereas systems that trigger downstream actions (e.g., molecular testing or additional review) may instead prioritize precision to reduce unnecessary follow-up.

This variability is also reflected in task-dependent baseline agreement levels, where endpoints such as mismatch repair immunohistochemistry loss show high concordance, more subjective tasks such as mitotic figure detection exhibit lower reproducibility even among experts, limiting the usefulness of universal target ranges for concordance or non-inferiority margins. Similarly, operational constraints vary by use case, with quality control or triage tools requiring minimal latency to maintain throughput, whereas non-urgent applications may allow longer runtimes in exchange for improved performance. As a result, validation should jointly consider diagnostic metrics (e.g., sensitivity, specificity, concordance), failure rates, and operational metrics (e.g., turnaround time), interpreted within the intended clinical context. In early deployment, a practical approach is to anchor expectations in published literature for comparable use cases and benchmark against similar systems in other institutions where available, refining thresholds as real-world evidence accumulates.

Although not addressed in detail here, individual tool generalizability across institutions remains an ongoing and significant challenge. At a slide level, variability in staining protocols, scanner hardware, and slide preparation can substantially impact algorithm performance, often necessitating site-specific adaptations or fine-tuning. However, an even greater challenge may lie in the wide-ranging institutional differences between hospitals themselves—including their infrastructure stacks, IMS and LMS systems, compute resources, clinical coding standards (e.g., stain code, diagnostic code), and reimbursement frameworks. Each hospital's setup, having evolved largely in isolation over many decades, often requires substantial rework of existing tools that may already be validated at a single site. This lack of standardization limits the potential for seamless “plug-and-play” deployment as tools transition between institutions. Notably, at each site tools typically begin in a Research Use Only mode until locally validated, because LDT status cannot be transferred across labs. Therefore, as these tools become increasingly deployed in clinical practice, establishing robust frameworks for assessing and adapting tools to diverse clinical environments will be essential to ensure safe, reliable, and effective broader deployment across healthcare systems. To that end, the Swiss Digital Pathology Consortium (SDiPath), has formed a Swiss Digital Pathology Algorithm Alliance (SDiPAA) working group, to foster inter-site communication and standardization of best practices.^m^

Relatedly, whereas we cannot speak authoritatively on CAP/CLIA requirements, our collaboration with a CAP-accredited institution to deploy and clinically validate computational pathology algorithms has provided some preliminary insight into how these frameworks compare. Although this work is still ongoing, it has highlighted that the fundamental principles of lab accreditation and validation remain broadly consistent across regulatory systems, even when operational details differ. In practice, whereas institutional structures, documentation formats, and procedural workflows may not map directly between settings, there is often a strong equivalence at the level of underlying responsibilities and required evidence. Specifically, regardless of jurisdiction, accreditation frameworks converge on a common set of expectations: defining the intended performance of a tool, demonstrating that performance under local conditions, and establishing processes for ongoing monitoring and quality assurance to ensure sustained performance over time. What differs primarily is not the conceptual validation strategy, but rather the administrative execution, required documentation granularity, and the distribution of responsibilities across institutional roles. From this perspective, the framework described here should be interpreted as an adaptation of well-established lab validation principles (as used for LDTs under ISO 15189-aligned systems) to computational pathology, rather than as a novel regulatory paradigm. This implies that most institutions do not need to build entirely new governance or quality systems, but can instead extend and repurpose existing structures to accommodate digital tools.

Whereas technical validation is essential, fostering user trust through transparency, education, and robust feedback mechanisms is equally critical. It is important to remember that the end users—primarily pathologists and, in some cases, lab technicians (e.g., during quality control)—must feel confident and comfortable when using these tools.^54^ This becomes especially vital as DP tools grow more sophisticated, moving beyond simple detection to complex tasks such as prognostic assessments or therapy response predictions based on image-based biomarkers. Ultimately, these users are responsible for signing-off on reports and integrating the tool's outputs into clinical decision-making in collaboration with their clinical partners, whether they be oncologists, surgeons, or other specialists. Ensuring their acceptance and confidence requires more than just delivering superior technology; it demands clear communication, comprehensive training, and ongoing opportunities for users to provide feedback. Only through building trust and transparency can these tools be successfully adopted in routine clinical practice.

Approaches to measuring training effectiveness, competency, and user acceptance must also account for practical constraints in routine clinical environments. Whereas formal assessment strategies such as structured surveys, proficiency testing, or periodic performance audits are conceptually straightforward, their implementation must avoid imposing excessive administrative burden, particularly in future settings, where clinicians may interact with large numbers of deployed computational pathology tools. Even relatively lightweight annual re-certification requirements could become operationally significant if scaled across many algorithms, highlighting the need for risk-proportionate, safety-preserving standards that provide meaningful oversight without becoming burdensome.

In parallel, more data-driven approaches may offer a complementary and less intrusive strategy for long-term monitoring of user performance and system impact. Under stable conditions, population-level diagnostic distributions (e.g., incidence rates of specific entities or diagnostic categories) are expected to remain relatively consistent over time, and meaningful deviations could serve as indirect signals for either performance drift or user-related variability. However, current hospital information systems are often not optimized for such analyses, as key clinical outcomes are frequently embedded in unstructured narrative reports rather than standardized, queryable fields. This limits straightforward statistical monitoring at scale. Future integration of structured reporting and advances in language models capable of extracting and normalizing diagnostic information from free text may enable continuous, low-burden assessment of both user behavior and system performance, supporting earlier detection of anomalies at both the individual and institutional level.

The need for strong institutional support and clear governance structures cannot be overstated when deploying such tools in clinical environments. Successful implementation relies not only on technical excellence but also on well-defined policies, oversight committees, and cross-disciplinary collaboration to manage risks, ensure compliance, and oversee ongoing performance monitoring. Institutional leadership must prioritize resource allocation, establish accountability frameworks, and foster a culture that embraces innovation while maintaining patient safety as the paramount concern. Without such comprehensive governance, even the most advanced tools risk underutilization, misuse, or unintended consequences, ultimately limiting their clinical impact and value.

An important consideration in implementing this framework is the size and staffing capacity of the institution. Whereas our institution is among the larger university hospital systems in Europe, we believe the framework remains feasible in smaller settings because it is not dependent on uniquely large teams, but rather on the presence of a limited set of essential competencies. Importantly, none of the steps in the framework are optional; instead, scalability is achieved through the consolidation of roles described in *Section 1.1* into a smaller number of individuals who collectively cover the required expertise. In a minimal configuration, the framework can be operationalized by approximately three core functions. First, a pathologist (combining the roles of *EP* and *RP* where necessary) ensures clinical relevance, defines use cases, evaluates algorithm performance on representative cases, and ultimately validates that outputs are safe, interpretable, and useful in routine diagnostic workflows. Second, an engineer or technical lead (combining *AR*, *DE*, and *IT* responsibilities) is responsible for algorithm adaptation, system hardening, integration into the local IT environment, implementation of testing and monitoring pipelines, and ensuring cybersecurity and infrastructure compatibility. Third, a quality/regulatory officer ensures compliance with internal governance standards and accreditation requirements, oversees documentation, coordinates audit readiness, and maintains alignment with regulatory expectations throughout the lifecycle of the tool.

In accredited labs, many of these competencies are already present, meaning that implementation often does not require introducing entirely new personnel but rather reorienting existing expertise toward computational pathology workflows. Nevertheless, as complexity increases, additional roles (e.g., lab management, departmental leadership, and dedicated IT support) become beneficial to ensure robustness, sustainability, and scalability. More generally, teams with greater disciplinary diversity are advantageous, as they enable cross-validation of assumptions, reduce blind spots in deployment decisions, and provide an internal peer-review mechanism for both technical and clinical aspects of the system.

## 4 Conclusion

We are at a pivotal moment in the evolution of DP, where research-driven tools are beginning to enter routine clinical workflows. This transition brings tremendous opportunities for innovation, learning, and improved patient outcomes. However, it also underscores the critical responsibility to ensure these tools are safe, reliable, and clinically sound. This manuscript, with its associated supplemental templates, has proposed a general framework for validating DP tools as LDTs, emphasizing patient safety at every stage. As the field matures, open sharing of methodologies, challenges, and best practices will be essential to accelerate development, streamline deployment, and maximize clinical impact. We encourage the broader community to contribute their insights so that, together, we can advance the safe and effective integration of these transformative technologies into everyday care.

## Ethics statement and patient consent

The project was reviewed and approved by the Geneva Cantonal Research Ethics Commission (Commission Cantonale d'Éthique de la Recherche, CCER) under approval number 2022–01929, dated February 2, 2023. All procedures were performed in compliance with institutional policies at Geneva University Hospitals (Hôpitaux Universitaires de Genève, HUG). The privacy and confidentiality of all patient data were strictly maintained throughout the study, and no identifiable personal information is included in this publication.

## Declaration of generative AI and AI-assisted technologies in the manuscript preparation process

During the preparation of this work, the authors used ChatGPT in order to improve clarity, succinctness, and ensure completeness. After using this tool/service, the authors reviewed and edited the content as needed and take full responsibility for the content of the published article.

## Declaration of competing interest

The authors declare that they have no known competing financial interests or personal relationships that could have appeared to influence the work reported in this article.

## Appendix A Supplementary data

Supplementary material: Practical Templates for Software Deployment, Validation, and Accreditation

## Footnotes

h https://www.sas.admin.ch/sas/en/home.html

i https://www.dakks.de/en/home-en.html

j https://www.cap.org/laboratory-improvement/accreditation/laboratory-accreditation-program

k https://eur-lex.europa.eu/eli/dir/1998/79/oj/eng

l https://eur-lex.europa.eu/eli/reg/2017/746/oj/eng

m https://sdipath.ch/swiss-digital-pathology-algorithm-alliance/
