# Supplementary material for: A guide for the deployment, validation, and accreditation of clinical digital pathology tools
Source: J Pathol Inform. 2026 Jul 28;23:100694. doi: 10.1016/j.jpi.2026.100694 (PMC13627954; doi:10.1016/j.jpi.2026.100694)
Supplement: Supplementary file 1 [file mmc1.pdf]

Appendix 1 - Software Specification, Responsibility, and Confirmation Plan

Before initiating the validation of a software tool, it is essential to clearly define and document the intended purpose, technical requirements, and assign responsibilities for each component of its lifecycle to qualified personnel. This document must align with Regulation (EU) 2017/746 of the European Parliament and of the Council on in vitro diagnostic medical devices, in particular Annex I – General Safety and Performance Requirements[i,25]

Required content:

1. Information regarding the requesting service or specialty – namely the Algorithm Researcher, and other accountable personnel (e.g., Laboratory Manager, Department Leadership, Quality Officer, Deployment Engineer, etc.).
2. Information about collaborating external laboratories, contact persons and development standards used (e.g., ISO 12207/29110, SWEBOK 3.0).
3. Tool description:
    a. Name and current version
    b. Brief description of intended purpose and use
    c. If a medical device, its classification under EU Regulation 2017/746 Annex VIII (Class A–D)
    d. Analysis type (e.g., diagnostic, prognostic, region detection, quality control)
    e. Result type (e.g., classification, segmentation, numeric value)
    f. Anticipated areas of performance improvement (e.g., time-gain efficiency, accuracy, interobserver variability)
4. Use Case Definition:
    a. Estimated daily analysis volume (e.g. Total number of slides per day)
    b. Number of slides *per case*? (e.g., 1 to 3, 5-20, 20+)
    c. Estimated percentage of positive cases, (if applicable)
    d. Criteria and specificity for slide identification (e.g., examination codes, staining, specimen description)?
    e. Required magnification for task completion?
5. Workflow integration:
    a. Description of result format (e.g. heatmaps, overlays, patch gallery)
    b. User-facing usability/ergonomic requirements, addressing how the results will be presented, integrated, and manipulated within the target software environment (e.g., IMS).
    c. Acceptable turnaround time (scanning to result display)
    d. Computation mode (e.g. precomputed vs reactive). Note runtime constrains.
    e. Slide review time and frequency: i.e., are the slides reviewed during a specific time of day?
6. Tool Functionality & Requirements:
    a. Minimum hardware and network specifications
    b. Define inputs and outputs as well as interfaces with other software, connecting this with **Appendix 3 Validation Plan Section 1.6.2**
    c. Software stack overview of major components (e.g. Docker, Flask, Pytorch, Spatilite). Smaller components and libraries are better described in an environmental file (e.g., requirements.txt with pip).
    d. List of software functions including expected input validity checks, sequence of operations and input-output relationships, responses to errors and abnormal situations, and effects of different parameters.
    e. Tool processing requirements, such as number of expected/needed concurrent executions and/or minimum number of (potentially concurrent) users, along with needed throughput on expected cases.
    f. Data storage schema, access policies and database structure.
    g. Archiving policies and auditing requirements for output.
    h. Design Constraints imparted by any security policies, internal guidelines, upstream tools, or project specific limitations.
    i. Additional software requirements (e.g., reliability, availability, security, maintainability, portability).
7. Data Management:
    a. Defining role-based access to patient data, results and software settings, aligning with ISO 15189, §5.10.2.
8. Software Life Cycle Management:

---

[i] https://eur-lex.europa.eu/eli/reg/2017/746/oj/eng

a. Define responsibilities in line with EU2017/746 Annex I, Ch II, §16.2 for the software's (a) maintenance, (b) failure management, (c) version tracking, (d) user support, and (e) decommissioning decision.
b. Including a long-term maintenance plan is recommended.
c. Define **Monitoring Plan**. If no calibration reference is available, an internal or commercial reference can be used periodically to verify consistent performance. When applicable, an EQA program can be employed.
d. Define a response plan in the event of a system, in line with requirements from ISO 15189, §5.10.3.

9. **Software Verification Plan**– a list of best-practice software development tests which should be conducted or a justification for their non-completion (e.g., unit tests, integrative tests, stress/load testing, reproducibility/reliability benchmarking tests) and a peer code review.

Before execution of the **Annex 3: Validation Plan**, the above items should be frozen, and then the items below should be validated as completed and signed-off on:

10. Software requirements and tests from **9. Software Verification Plan** were validated and functionally verified by the laboratory before and after implementation.
11. Software Documentation completed including insuring (a) The code is documented, (b) there is a text file named README, which serves as a label within the meaning of EU Regulation 2017/746 (Annex I, Art. 20.2), accessible on the intranet, and its location, and (c) a user manual with its location.
12. This user manual should be confirmed to contain, in line with EU2017/746 Annex I, Ch III, §20.4.1, and **Annex 5**: **Standard Operating Procedure**:(a) the name of the software, (b) description of its purpose and use, (c) restrictions and warnings for the user, (d) specifications and expected performance, (e) installation and configuration procedure, (f) a procedure for verifying that the software is installed and functioning correctly, (g) publication date, (h) version number, and (i) a list of minimum hardware and network requirements.

Lastly, a final statement of conformity with the requirements, and approval of the various responsible stakeholders including, Laboratory Manager, Deployment Engineer, Quality Officer, and Department Leadership.

Appendix 2 - Analysis Of Risks Impacting Work Processes During Major Modifications

Example of risk a management strategy document following ISO 15189 § 4.14.6. It is used to identify potential risks and define mitigation strategies. In addition, it helps inform the validation plan to ensure risks are properly mitigated.

This document is typically required in cases of (a) installation of analytical equipment, (b) introduction of a new inter-laboratory workflow, (c) laboratory relocation, or (d) changes in the laboratory's assessment procedure.

First, high-level risk categories are defined, which may include items like:

1. General Risks:
    a. Misuse of the system
    b. Interaction with other systems
    c. System failure or malfunction
2. Information Security Risks:
    a. Data transmission (internal network and/or external web interfaces)
    b. Unauthorized access or modification of data.
    c. Data loss or corruption.

Once a high-level risk assessment is established, a detailed risk table is developed, individually listing specific risks:

a) The exact scope/nature of the risk
b) The impact of the associated risk
c) Frequency Value (see **Table S1**)
d) Severity Value (see **Table S2**)
e) Non-Detection Value (see **Table S3**)
f) Pre-amelioration risk score, which equals: Frequency value * Severity value * Non-detection value
g) Determination if this risk is acceptable, typically categorized into 3 levels:
    a. Acceptable risk: No further action required.
    b. Medium risk: Awareness of risk but likely no intervention needed
    c. High risk: Mitigation actions need to be taken accordingly
h) Action plan for addressing risk
i) Post-amelioration risk score, computed similar to (f), now assessing residual risk after completing (h) action plan
j) Deadline: when or at what stage the risk mitigation needs to be implemented – notably this can as well contain the notion of "continual" risk mitigation, to indicate that monitoring is required
k) Responsible personnel for addressing specific risks.

The values for risk assessment are based on established conventions, reproduced below for completeness[ii,35]:

Frequency

Frequency Type	Likelihood	Value
Non-existent - No known occurrence	1/10,000	1
Low - Possible, no existing data	1/5,000	2-4
Moderate - Documented, but uncommon	1/200	5-6
High	1/100	7
Documented and frequent	1/50	8
Very high	1/20	9
Near certain error	1/10	10

Table S1. An expectation, based on literature review or laboratory experience, of the likelihood of the particular risk to occur. Each level of increasing frequency comes with an increasing value, imparting a stronger need in having an associated mitigation strategy.

[ii] https://www.suva.ch/fr-ch/download/document/documents-de-formation-sur-l-evaluation-et-l-attenuation-des-risques/standard-variante--66099.F

Severity

Severity Type	Value
Minor annoyance of system	1
Minor Systematic Problem	2-3
Major Systematic Problem	4-5
Minor Patient Injury	6
Major Patient Injury	7
Quality, safety problem	9
Terminal illness or Death of the patient	10

Table S2. This table assigns a value for the severity if the associated risk does indeed come to fruition. As expected, minor annoyances receive a small value, while those potentially leading to the death of a patient receive the highest values. Similar to frequency, a higher severity value contributes to a stronger need in having an associated mitigation strategy[iii,27].

Non-Detection

Detection capability	Value
The detection system is infallible	1
Detection system in place but not infallible	5
No detection system	10

Table S3 This value determines the likelihood of a mistake in the system being successfully detected. For example, this can be the difference of an algorithm working "in the background", with no user facing feedback (e.g., a screening test), versus a computer aided diagnostic tool, wherein the pathologist interprets raw data and errors are more organically captured during routine tool usage.

Once each identified risk is assessed for both frequency (likelihood) and severity (impact), the corresponding values are mapped onto a "risk matrix". The matrix provides a structured way to determine the **overall risk level**, which in turn informs decision-making regarding acceptance, mitigation, and monitoring requirements.

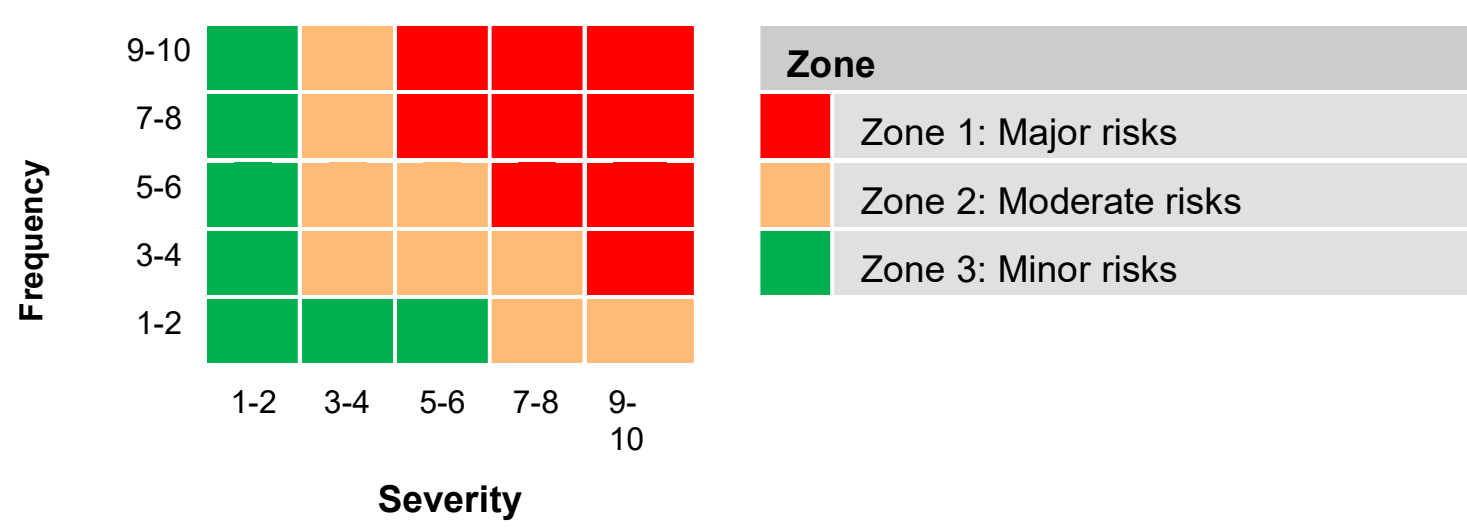

[iii] https://eur-lex.europa.eu/eli/reg/2017/746/oj/eng

Appendix 3 - Validation Plan and Report

This document is the core report for validating a tool in a digital pathology workflow. Sections are progressively completed in unison with the phases in **Figure 1**. The finalized report is co-signed by all key stakeholders (Deployment Engineer, Laboratory Technicians, Quality Officer, Laboratory Manager, and Departmental Leadership), to confirm that the tool has been validated for routine clinical use.

This document is typically structured in two major sections, (a) the **Planning** section outlines specific methodology, test procedures, and acceptance criteria for validation, while (b) the **Report** section summarizes the resulting outcomes.

1 Planning Section

1.1 Introduction And Objectives

This section describes the precise task, and tool performance, which needs to be demonstrated. To ease interpretability and re-use, categories of common validation tasks can be created (e.g., validate new algorithm, verify new scanning technique, comparison of two established techniques). Where applicable, include references to publicly available information which may provide an expected performance baseline. This supports the objective of demonstrating that the tool produces reliable and reproducible results, performing comparably to established methods. If the verification relates to an already validated technique, the initial validation report should be referenced.

1.2 Budget

A section describing approximate total costs and how the validation/accreditation process will be funded is frequently required by administrators.

1.3 Personnel Concerned

Documentation of different roles and responsibilities is critical for traceability throughout the validation process. One can align these with the roles introduced in **Section 1.1** of the main text, and can be categorized into: (a) Project management, (b) Execution, (c) Reporting, and (d) Approval.

The QA Officer ensures that all tests, results, and reporting comply with current regulations. Notably, this person does _not_ have to be an expert in DP. Instead, their expertise lies in applying and verifying "*critical criteria"* — essential requirements or standards required to ensure the safety, quality, and regulatory compliance of a product or process.

1.4 Equipment And Technique

1.4.1 Equipment – Location And Certification Markings

Information regarding the provider, location, and identification numbers of the equipment and software employed within the validation process are noted. Importantly, the clinical validation process only validates the tool for usage *on those* specific pieces of equipment, unless they are part of a collection of similar items. For example, one would mark down the server which is running the algorithm, but would not need to individually list and validate/accredit every terminal which uses the software/output provided by that server.

For clinical validation of a tool, it is essential to evaluate its performance in real-world clinical analyses, ensuring users have reliable, robust tools that function consistently regardless of where or how the tool is executed. To meet this requirement, the various combinations of equipment and software that are expected to be involved in the routine work must be marked, tested, and evaluated for cross-combination consistency. Additionally, if any of the equipment or tools have IVD or CE markings, their associate numbers should be marked down for reference.

1.4.2 Quality Assurance Of The Laboratory's Equipment And Software

A checklist can be provided to confirm the laboratory is currently compliant and suitable for conducting a validation process:

(a) Ensure supplier/manufacturer/provider is correctly listed in the "Register of suppliers and manufacturers". This registry is an accreditation document verifying suppliers meeting all criteria for medical equipment suppliers and/or is registered in the hospital's supplier database.

(b) Availability of "Installation Qualification (IQ) and Operational Qualification (OQ)" reports, certifying devices were installed according to the manufacturer's specification and operates according to its intended functions within specified limits.

(c) Equipment warranty status

(d) Equipment regularly undergoes preventive maintenance

1.4.3 Technique

Provide a descriptive name of the tool intended for validation, as well as a link to the completed versions of the **Appendix 1: Software Specification, Responsibility, and Confirmation Plan** and **Appendix 4: Standard Operating Procedure (SOP).**

1.4.4 Material

Indicate the specific type of material which will be used in the validation process., for example, slides of (a) frozen tissues, (b) formalin-fixed paraffin embedded tissues, (c) classical histochemical stains (e.g. HE, PAS), (d) cell smears, (e) immunohistochemistry stains, or (f) digitized images. Multiple categories may be relevant and selected.

1.4.5 Type Of Method/Technique

Describe how the technique was developed, including relevant specifications, definitions, methods and options. This clarifies the origin of the tool and its associated baseline performance metrics, which serve as references for verifying correct operation—whether they are supplier-defined and should be followed more strictly, or internally developed and adapted from the literature to reflect site-specific conditions.

Type	Definition
Commercial	Applied 100% according to the supplier's instructions
Standard	Applied 100% according to the referenced standard
Literature	Applied 100% according to the literature
Internal	Method/technique developed by the laboratory, or a combination/modification of Commercial, Standard, and Literature based techniques.

Table S4 An example definition table for assigning the origin of the particular technique.

1.5 Sampling Of Slides

This section defines the anticipated cohort used for the validation. It should include:

- The expected number of slides to be evaluated
- The clinical sectors/specialties (e.g., gynecology, gastroenterology)
- A descriptive explanation of the inclusion criteria for cases selected for validation, along with the intended distribution of outcomes (e.g., percentage of positive vs. negative cases, and/or proportion of categorical, discrete, and continuous data types).
- A table of specific case IDs

1.6 Validation Steps

For planning purposes, an estimated implementation date for the tool is provided, along with a projected timeline for notifying Quality Assurance and other relevant departments (e.g., IT), to enable proper coordination and resource allocation at departmental level. The validation steps are then described at a high level.

1.6.1 Acceptance Criteria

Provide a list of performance acceptance criteria, which for subsequent versions of tools may contain statements like "Demonstrated specificity of the new version superior to the performance of the current version by at least 5%".

1.6.2 Connectivity and Data Transmission

Determine whether equipment connection/integration tests are required, to ensure data transfers are complete and compliant (e.g. analysis number, date, names, results are all present and in appropriate formats, see **Appendix 1.6**.

1.6.3 Performance

Verify the tool performs and operates within its predefined performance specifications, see **Appendix 1.6**.

1.6.4 Validation of Technique

A table is produced with the minimum performance requirements of the tool, based on either a supplier provided document or metrics determined by literature review (with those manuscripts cited). Metrics may include acceptable ranges for sensitivity, specificity, kappa, precision, along with comparison and reproducibility between scanners, measurement uncertainty, acceptability of results in the absence of an established external quality assessment (EQA) program, and confidence level of pathologists. These latter items are discussed in more detail below.

1.6.4.1 Reproducibility

If the laboratory uses multiple scanners, models, or brands, a reproducibility study should be performed to ensure consistent output across them. Concordance rate, continuous metrics (e.g., quantitative measurement differences), and visual assessment results should be reported.

1.6.4.2 Measurement Uncertainty

Measurement uncertainty is defined by ISO15189 as "*non-negative parameter characterizing the dispersion of the quantity values being attributed to a measurand, based on the information used*". In practice, this can be described as, e.g., the standard deviation expected, considered acceptable, and verified and compared to the quantitative metrics table produced in **Section 1.6.4**.

1.6.4.3 Acceptability Of Results In Absence Of An Established EQA Program

In the absence of a formal EQA program, confidence in validation should be established through internally controlled domain experts defining the ground truth.

1.6.4.4 Confidence Level Of Pathologists

To assess experience of the end user (e.g., pathologists), the tool is integrated temporarily into their routine workflow, and evaluated alongside unaided/conventional task completion.

The user then evaluates the tool's accuracy, usability, and perceived associated risks (e.g., consistently identified false positives), and record their feedback. Key evaluation criteria may include the accuracy compared to conventional methods, the tool's ease of use and integration into existing workflows, and the risks of misinterpretation, manipulation errors, and missed critical areas on digital slides.

The feedback forms can collect both quantitative ratings and qualitative feedback, which is compiled to identify trends. The resulting summary report may include recommendations for improving the tool, training needs, and/or risk mitigation strategies.

1.6.5 Software To Use

Here the version of all the software, hardware, and infrastructure are noted down for reproducibility purposes. Commonly one may list things as (a) scanner software, (b) viewer software, (c) software managers, and (d) other software.

1.7 Acceptance Criteria

Finally, remaining notes on the acceptance criteria takes place.

Acceptance Criteria: While a number of different metrics were proposed for measurement and targeting above in **Section 1.6.4** , it is possible that a tool may still be accepted if only a subset of those criteria are met. This section ultimately defines what requirements are needed for acceptance, but this may be as simple as simply saying 100% compliant with items listed above.

Discrepancy Handling: indicating if discrepancies will be reported to provide insight into areas where pathologists may need to exercise caution.

Deviation planning: how deviations from the above plan will be addressed, for example, they may be summarized and presented in such a way as to highlight any non-conformities, with any deviation from the plan or any result outside the acceptance criteria requiring justification and investigation using a deviation/non-compliance form. Explicitly stating if these must be approved and closed should also be noted.

1.8 Approval Of The Validation Plan

Finally, with all documents completed, reviewed, and approved, they are signed off by the Laboratory Technician, Laboratory Manager, and Quality Officer.

2 Report

Once the plan has been executed, a report is produced which summarizes the findings to enable a decision for validation acceptance. In such a report, the following sections can be excepted.

2.1 Introduction And Objectives

A short high-level overview contextualizing the rational and objectives for the validation/accreditation of this tool

2.2 Progress Of Validation

A precise list of the actual validation having taken place, e.g., the number and type of slides, and how many slides were used for comparison against a previous tool or version of this tool if applicable. This can include an explanation, or any noted differences, from the expected validation plan approved in **Section 1.**

2.3 Staff Performance Review

An assessment after the validation process to assert if the necessary competencies exist in the laboratory to use the tool in routine practice, or if additional training need to take place, including a reference to that training plan.

2.4 Evaluation Of The Risks

A discussion of previously identified risks (from **Appendix 2**), as well as any new risks discovered during tool validation. Ideally, these risks have already been addressed—either through improved tool design or through safety measures outlined in the Standard Operating Procedure (SOP). Users should be made aware of these potential error modes and trained on how to prevent them during onboarding.

2.5 Results

2.5.1 Software Used

Any deviations from the planned versions for the e.g., tool, image viewer, or other software are noted down.

2.5.2 Verification And Acceptance Criteria Of Hardware And Software

Reporting results to **Section 1.6.1**, this section answers the question: were all associated hardware and software used during this validation within compliance?

2.5.3 Validation Of Computer Connection And Transmission

Reporting results to **Section 1.6.2**, this section answers the question: was the necessary and correct output from the tool successfully transmitted to its downstream data storage locations?

2.5.4 Software Performance Validation

Reporting results to **Section 1.6.3**, Here we confirm if the software worked as expected without e.g., crashing, or any other unexpected anomalies.

2.5.5 Validation Of The Technique

To begin, the participants (e.g., pathologists) and sectors (e.g., which organ groups) that actually (versus planned) participated in the generation of the results are listed for traceability.

2.5.5.1 Performance Areas

The results obtained during the execution of the validation, including any deviations from the planned process (e.g., actual sample counts), should be documented. This section serves as the completion of the plan outlined in **Section 1.6.4,** and includes the recording of relevant performance metrics. Any deviations from the original plan must be clearly noted, along with confirmation that the tool and environment were set up and operated in accordance with the manufacturer’s instructions.

2.5.5.2 Comparison And Reproducibility Between Scanners

Reporting results to **Section 1.6.4.1**, stating if the resulting test yielded acceptable results.

2.5.5.3 Measurement Uncertainty

Reporting results to **Section 1.6.4.2**, with a final statement comparing the expected versus measured certainty of the results. Any noted uncertainty in the form of variations in image quality, algorithm performance under different conditions, and potential discrepancies between tool-generated and manual results should be noted.

2.5.5.4 Acceptability Of Results In The Absence Of An Established EQA Program

Reporting any relevant results to **Section 1.6.4.3**.

2.6 Validation Statistics By Pathologists

A summary of the survey results provided by the uses after having tested out the tool during their clinical routine, essentially a reporting of data collected in **Section 1.6.4.4.**

2.7 Conclusion

A reporting against the acceptable criteria put forth in **Section 1.6.7**. This is essentially a listing of the required Parameters, the laboratory acceptance criteria levels, those obtained by the tool, and if these are acceptable. One then simply provides a checkbox indicating if the validation conforms or not to the needed specifications.

2.8 Approvals Of The Validation Report

Lastly, the validation report is signed and dated by the various translational group members which engaged in its completion. These likely include personnel such as the Laboratory Technician, Laboratory Manager, Head of the Department, and Quality Officer.

Appendix 4 - Production Deployment Checklist

During deployment several factors should be verified, typically via a checklist:

1 Coordination

Has the Quality Manager been informed, via the appropriate document, whether the developed technique is:

- A new technique requiring referencing
- A modification of an existing technique
- A replacement for a previously validated technique.

Have associated costs been identified and (if applicable) reimbursement/coding systems updated accordingly?

2 Documentation

Are the necessary documents uploaded into an audit-approved repository? These documents may include at minimum:

- Standard Operating Procedures
- Signed validation report

3 Personnel

Has technical training taken place? Confirm there is a plan in place for detecting when retraining is needed, or that no plan is required. Has the **Competence Register** been updated?

4 Instruments

Confirm presence, availability, and accessibility of documentation (i.e., either a physical binder or a digital folder) regarding: validation raw data, validation files, instructions, and training documents

Confirm that a regular maintenance routine has been documented and implemented. For software tools this may include critical items which do not directly impact performance and thus would not trigger a revalidation (e.g., security patches).

5 Monitoring

Confirm existing and implementation of a monitoring program (see **Appendix 1.8c**).

6 Validation Report

Verify the Validation report been generated, reviewed, and signed before putting the tool into production.

7 Final Dataflow Verification

Ensure that the dataflows are ready between laboratory applications, software, and downstream processes as needed. This may include verifying registration of tools in the IMS, confirmation of appropriate port white listing on servers/routers, and appropriate user permissions for access of results are in place.

8 Report

Has the total Package for this tool been verified to ensure completeness, and ensure any/all “N/A” or “Subject to conditions” are appropriate?

9 Authorization

Obtain signatures by Laboratory Technician, Laboratory Manager, and Quality Manager.

Appendix 5 - Standard Operating Procedure Template

A Standard Operating Procedure (SOP) for a tool typically includes the following sections:

1. Title Page containing:
    - SOP title
    - SOP number
    - Version number
    - Date of creation/revision
    - Approval signatures and dates
2. Purpose
    - Description of the SOP's purpose
    - Explanation of why the SOP is needed
3. Scope
    - Definition of the scope of the SOP
    - Specific software tool(s) covered
    - Departments or personnel to whom the SOP applies
4. Definitions and Acronyms
    - Explanation of any terms, acronyms, or abbreviations used in the SOP
5. Roles and Responsibilities
    - List of personnel roles involved
    - Specific responsibilities of each role related to the tool
6. References
    - List of related documents, manuals, regulations, or standards referenced in the SOP
7. Procedure
    - Detailed step-by-step instructions on how to use the tool, in particular:
        - Setup, installation, and initial setup
        - Procedures for accessing and logging into the software
        - Overview of the software interface, navigation, and main features
        - Guidelines for data entry and management, including storage
        - Instructions on generating and interpreting reports
        - Procedures for software updates and maintenance
        - Steps for backing up data and recovering it in case of failure
8. Safety and Compliance
    - Guidelines for ensuring patient safety and data security
    - Compliance with relevant medical and regulatory standards (e.g., HIPAA)
9. Troubleshooting
    - Common issues and their solutions
    - Contact information for technical support
10. Training and Competency
    - Description of training requirements for users
    - Procedures for ensuring user competency
11. Audit and Review
    - Procedures for regular review and auditing of the SOP
    - Criteria for updates and revisions
12. Document Control
    - Procedures for managing SOP versions and updates
    - Distribution list and access control
